# New Diagnostic Assays for Differential Diagnosis Between the Two Distinct Lineages of Bovine Influenza D Viruses and Human Influenza C Viruses

**DOI:** 10.3389/fvets.2020.605704

**Published:** 2020-12-11

**Authors:** Faten A. Okda, Elizabeth Griffith, Ahmed Sakr, Eric Nelson, Richard Webby

**Affiliations:** ^1^Department of Infectious Diseases, St. Jude Children's Research Hospital, Memphis, TN, United States; ^2^Veterinary Division, National Research Center, Cairo, Egypt; ^3^Department of Chemical and Therapeutic, St. Jude Children's Research Hospital, Memphis, TN, United States; ^4^Department of Business Administration and Management, Dakota State University, Madison, SD, United States; ^5^Veterinary & Biomedical Sciences Department, Animal Disease Research and Diagnostic Laboratory, South Dakota State University, Brookings, SD, United States

**Keywords:** influenza D viruses, influenza C viruses, differential diagnosis, peptide ELISAs, blocking ELISA, diagnostic assay

## Abstract

Influenza D virus (IDV), a novel orthomyxovirus, is currently emerging in cattle worldwide. It shares >50% sequence similarity with the human influenza C virus (HICV). Two clades of IDV are currently co-circulating in cattle herds in the U.S. New assays specific for each lineage are needed for accurate surveillance. Also, differential diagnosis between zoonotic human influenza C virus and the two clades of IDV are important to assess the zoonotic potential of IDV. We developed an enzyme-linked immunosorbent assay (ELISA) based on two different epitopes HEF and NP and four peptides, and fluorescent focus neutralization assay to differentiate between IDV bovine and swine clades. Calf sera were obtained, and bovine samples underwent surveillance. Our results highlight the importance of position 215 with 212 in determining the heterogeneity between the two lineages. We needed IFA and FFN for tissue culture–based analysis and a BSL2 facility for analyzing virus interactions. Unfortunately, these are not available in many veterinary centers. Hence, our second aim was to develop an iELISA using specific epitopes to detect two lineages of IDVs simultaneously. Epitope-iELISA accurately detects neutralizing and non-neutralizing antibodies against the IDV in non-BSL2 laboratories and veterinary clinics and is cost-effective and sensitive. To differentiate between IDVs and HICVs, whole antigen blocking, polypeptides, and single-peptide ELISAs were developed. A panel of ferret sera against both viruses was used. Results suggested that both IDV and ICV had a common ancestor, and IDV poses a zoonotic risk to individuals with prior or current exposure to cattle. IDV peptides IANAGVK (286–292 aa), KTDSGR (423–428 aa), and RTLTPAT (448–455 aa) could differentiate between the two viruses, whereas peptide AESSVNPGAKPQV (203–215 aa) detected the presence of IDV in human sera but could not deny that it could be ICV, because the only two conserved influenza C peptides shared 52% sequence similarity with IDV and cross-reacted with IDV. However, blocking ELISAs differentiated between the two viruses. Diagnostic tools and assays to differentiate between ICV and IDV are required for serological and epidemiological analysis to clarify the complexity and evolution and eliminate misdiagnosis between ICV and IDV in human samples.

**Graphical Abstract:**
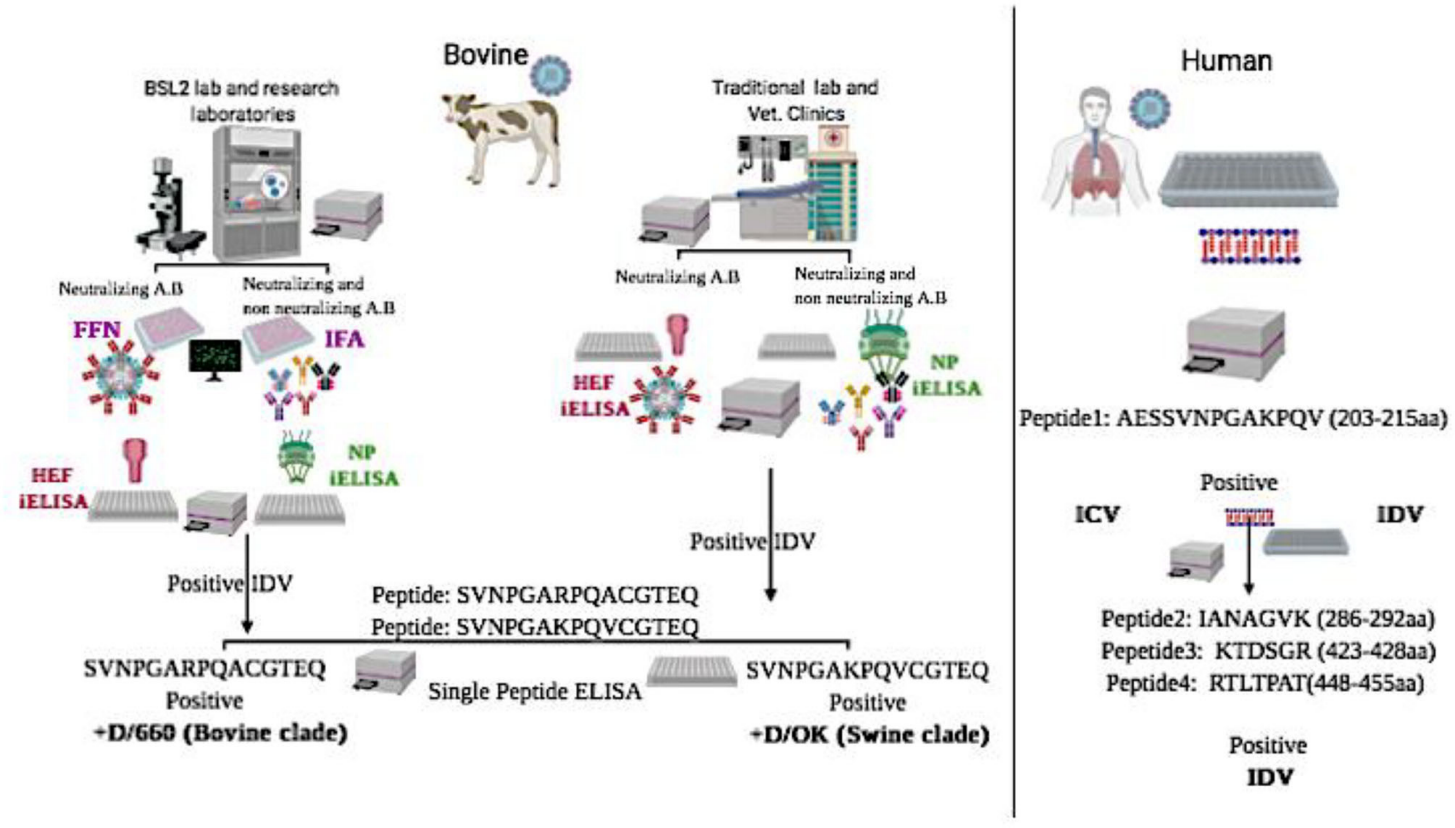
Graphical Abstract represents schematic flowchart for differential diagnosis between the two clades of IDV in bovine samples and between IDV and HICV in human samples using new diagnostic assays:

*NP-iELISA and HEF-iELISA for detection of IDV in non-BSL2 laboratories in bovine samples.

*Immunofluorescence and neutralization assays for detection of IDV in bovine samples.

*Peptide ELISA for differentiation between the swine and bovine lineage of IDV in bovine samples.

*Blocking ELISA and a panel of peptide ELISAs for differentiation between IDV and human ICV in human samples.

## Introduction

Influenza viruses (IVs) are a public health threat, as they are associated with a high rate of morbidity and mortality every year (1). Although both influenza A viruses (IAVs) and influenza B viruses (IBVs) cause annual epidemics in human populations, IAVs have been associated with several pandemics in past decades due to their wide host range and cross-species transmission (2, 3). As the host range of IBVs and influenza C viruses (ICVs) is limited, their mutational capacity is also limited, so they do not cause pandemics (1). IDV is a novel IV detected in cattle and swine and is antigenically and biologically distinct from the human ICV [2]. Several studies suggest that bovine species is the natural host reservoir, and IDV is possibly involved in the bovine respiratory disease complex (4).

Serological studies show the presence of IDV in beef cattle at least since 2004 (5). Bovine species are the main reservoir for IDV. However, young weaned and immunologically naive calves are very susceptible to IDV, as there is a reduction in maternal antibodies from birth to 6 months (6). IDV seroprevalence is ~62% in cattle, alone or with other respiratory viruses, that could influence the severity of respiratory syndrome disease in cattle (7). Moreover, serological studies on small ruminants in different US states report that 13.5 and 13.3% of sheep and goat farms, respectively, have IDV exposure (8). Calves weaned and co-mingled play a critical role in circulation and transmission of IDV, and neonatal calves acquire maternal immunity against IDV within the first 24 h through colostrum. However, this declines within 6 months (9, 10), as the passively acquired IgG has a half-life of 21.2–35.9 days (11). Enzyme-linked immunosorbent assays (ELISAs) and virus neutralization assays are considered accurate and rapid serological and diagnostic assays in veterinary and human medicine (12).

Common diagnostic tests for IVs include direct detection of virus attachment to sialic acid–linked receptors on red blood cells by the hemagglutinin assay (HA), or by detecting virus-specific neutralizing antibodies that inhibit virus binding to RBCs by using hemagglutination inhibition (HI), an agar gel precipitation test (AGP), or the micro-neutralization (MN) assay, an ELISA-based virus neutralization test (13, 14). Both the HA and HI are commonly used, but different IVs show variations in the avidity to RBCs (15). In a recent study, several IVs showed low binding to RBCs, and low avidity of some recent IVs has caused major issues due to inconsistent HI tests, especially for evidence relating to antigenic drift or determining changes related to the IV vaccine (16). Also, the HI test is labor intensive and requires many standardizations (17). Disadvantages of the agar gel precipitation test are low sensitivity and high time consumption (18, 19). The MN assay is considered more sensitive than the HI or agar gel precipitation tests, but it requires further standardization for routine evaluation of vaccine efficacy (20). HI requires reference sera specific for each virus for each test performed (14). HI can detect antibodies that can block the binding of viral hemagglutinin to receptors on RBCs, However, the MN test is a tissue culture–based assay to identify functional antibodies for hemagglutinin to prevent infection of cells (13, 21).

Reverse-transcription PCR detects viral nucleic acids and is commonly used to diagnose IDV (4). It is a highly sensitive assay for virus identification, but has low efficacy in large-scale surveillance, due to early clearance of viral nucleic acids (22). Given that two lineages of IDV are not cross-reacted using the HI test but they showed low or no HI titer to each other (4), an accurate ELISA test based on conserved antigen specificity for IDV, along with a fluorescence-based neutralization assay, is required for effective IDV surveillance. Moreover, new diagnostic assays specific for each IDV lineage are needed to obtain critical information about viral evolution and vaccine efficacy (12). Highly conserved proteins in the genus of IVs include nucleoproteins (NPs), with only 20–30% of intergenic homologies (4). Therefore, we aimed to develop a highly sensitive and specific ELISA based on two different epitopes NP and HEF for serological studies and four different peptides to differentiate between the two circulating lineages of IDV. We also planned to optimize a quick and accurate fluorescent focus neutralization (FFN) assay using a high-avidity-binding polyclonal antibody specific for the whole viral antigen.

Influenza D virus (IDV) can cause respiratory disease in pigs and cattle and is closely related to human ICV (23). IDV shares 50% amino acid sequence identity with ICV, lacks the neuraminidase gene, and has seven genes instead of eight seen in IAV and IBV (4). Based on the National Center for Biotechnology Information analysis, Sequence similarity between ICV and IDV is as follows: PB2 gene, 58.5%; PB1 gene, 66.7%; P3 gene: 57.29%; HEF gene: 55.7%; NP gene: 51.4%; NS gene: 47.5%; and P42 gene; 52.4% (Table 3 and **Figure 5**). ICV infection is characterized by mild clinical respiratory illness with a low frequency of infection, commonly in young and elderly individuals (24). IDV can infect ferrets, but has not transmitted from human to humans to date (4, 5). ICV has a global distribution in children (25), and is reported in pigs (26), dogs (27), and camels (28). In contrast, IDV has been reported in swine (23), cattle (4), goat (8), and equine (29) species. IDV has shown serological reactivity with plasma collected from human exposed to cattle (30). In 2017, IDV was detected in rectal swabs, raising the concern that it could replicate in the intestinal tract as IAV and IBV do (31). ICV lacks an open channel due to a salt bridge interaction. However, IDV has an open channel that can accommodate a diverse range of glycans, which can contribute to broad cell tropism. Some genetic and antigenic IDV lineages do not reassort with ICV or IDV or cross-react with antibodies against some human ICVs (32). In our study, we optimized IDV and ICV blocking and peptide ELISAs with known antibody panels specific to each virus, which can be used to detect and distinguish between ICVs and IDVs in human serum samples.

## Materials and Methods

### Cell Culture and Virus Production

Madin-Darby canine kidney (MDCK) cells were cultured in DMEM supplemented with 10% fetal bovine serum (FBS) and 1% penicillin and streptomycin. Influenza D/bovine/Oklahoma/660/2013 (D/660 or bovine clade) and D/swine/ Oklahoma/1334/2011 (D/OK or swine clade) were previously isolated from bovine or swine showing symptoms of respiratory disease (23). Influenza C/Human/Johannesburg-1/1966 were obtained from St. Jude Children Research Hospital. Viruses were propagated by infecting confluent MDCK cells at a multiplicity of infection of 0.01, and then incubated at 33°C with ~5% CO_2_ for 5 days, using virus growth/maintenance media and DMEM with 0.1 μg/mL TPCK-treated trypsin. After 5 days, infected cell cultures were frozen and thawed thrice, and the supernatant was centrifuged at 500 × *g* for 15 min at 4 degree to remove cellular debris. Virus titer was determined by using MDCK cells according to the Reed and Meunch method (33).

### Serum Samples

Approximately 500 randomly selected bovine serum samples were collected (South Dakota State Animal Disease Research and Veterinary Diagnostic Laboratory) mostly 6-month-old calves. Sample from 1 year to 2 years old bovine, bovine sera from different seasons, and ferret sera (from previous experiments) were supplied by St. Jude Children's Research Hospital.

### Hemagglutination Inhibition Assay

The HI assay was run in bovine serum samples as per World Health Organization standard manual (5). Samples were pre-treated with receptor-destroying enzyme (Denka 261 Seiken, Chuo-ku, Tokyo, Japan). The HI assay was performed using 1% turkey RBCs (Lampire Biological Laboratories, PA, USA). Serial 2-fold dilutions of serum samples were tested in duplicate. Titers were expressed as reciprocal of the highest serum dilution of serum yielding complete hemagglutination. All samples were assayed in three separate experiments, and mean antibody titers were calculated.

### Indirect Immunofluorescence Assay

All bovine samples were tested by immunofluorescence analysis (IFA) developed and previously optimized (12) and classified as positive or negative.

### Preparation of Antigens Used for ELISA

#### Bacterial Expression and Purification of the IDV Nucleocapsid Protein (NP)

A full-length IDV NP gene sequence of the IDV strain D/bovine/Ok/660/2013 (GenBank: KF425663.1) was previously synthesized and used (12). Purified proteins were run on a western blot (WB) using anti-rabbit IDV polyclonal antisera previously generated in our laboratories.

#### Bacterial and Mammalian Expression and Purification of IDV Hemagglutinin Esterase

A recombinant plasmid with the synthetic truncated sequence of the region from 400 to 1,665 of the HE sequence of the IDV strain D/bovine/Ok/660/2013 (GenBank: KF425662.1) with the addition of a 3′ 6 × -His tag by GeneArt® Gene Synthesis (GeneScript, Piscataway, NJ, USA) was subcloned in the Pet28 system for bacterial expression as reported previously (34). Plasmid DNA of the HEF-truncated sequence (60-400) and the mutation at amino acid site 212 were linearized by digestion with restriction enzymes *Bam*H1 and *Xho*1 and then cloned into the pcDNA3.1 mammalian expression vector.

#### Whole-Virus Antigen

Sucrose-purified/ultraviolet-inactivated IDV strain D/660, C/OK, and HICV strains were used as a whole virus purified control antigen for IFA, WB, and ELISA. Trypsin-treated cleavage viruses were used as a control for HE cross-reactivity.

#### Preparation of Antibody Panels

The panel of antibodies included ferret α-C/VICTORIA/1/2011, ferret α-Johannesburg-1/1966, ferret α-D/OK/1334/2011, and ferret α-D/OK/660/2013, which were generated at St. Jude Children's Research Hospital (5). Experimental bovine α-D/OK/660/2013 and Experimental D/bovine/Mississippi/C00046N/2014 and D/bovine/Mississippi/C00013N/2014 were kindly provided by Dr. Xiufeng Wan and Dr. Lucas Ferguson, Mississippi State University. Convalescence-positive bovine D, rabbit α-D/OK/1334/2011, rabbit Dα-D/OK/660/2013, and negative control were prepared in South Dakota State University.

#### Peptide Design to Determine Heterogeneity Between the Two Lineages of IDV in Antibody Recognition

We designed four different peptide covers for amino acids K212 and R212 in both lineages. Four peptides were synthesized by Genscript. Four different peptides, SVNPGA***R***PQ***A***CGTEQ (206-220aa), SVNPGA***K***PQ***A***CGTEQ (206-220aa), SVNPGA***K***PQVCGTEQ (206-220aa), and SVNPGA***R***PQVCGTEQ (206-220aa), covering the region of position 212, were used. In the D/OK lineage, the K is in position 212 and the V in position 215, whereas in the D/660 lineage only R is in position 212 and A in position 215.

#### Preparation of Polypeptides and Single Peptides to Differentiate Between ICV and IDV

Based on the alignment between the HEF of ICV and IDV, we selected four different conserved peptides for each virus that were different in amino acid sequences. Amino acid sequences ranged from 7 to 16 amino acids (**Figure 5**). A combination of equal concentration of each peptide was used as a polypeptide antigen. For optimization, a panel of antibodies specific for ICV and IDV was used. The ICV sequence was used as antigen (which did not include the influenza D sequence) to reflect the antigenicity of ICV. Then, using the ICV sequence as antigen and IDV sequence as negative control, synthetic peptides were purchased (GenScript). Peptides were chosen based on conserved amino acid differences in the HEF protein. RTDKSNSAFPRSAD (74–87 aa), in which its conserved but like IDV 58%. GSRKESGGGVTKES (484–497 aa). We did not find conserved peptides specific to ICV, and they did not share any amino acids with IDV. For IDV, we found four conserved peptides different from those in ICV: AESSVNPGAKPQV (203–215 aa), IANAGVK (286–292 aa), KTDSGR (423–428 aa), and RTLTPAT (448–455 aa) (**Figures 4A**, **5**).

#### Cross-Reactivity Between the Two IDV Lineages

Rabbit polyclonal primary antibodies (IgG) against IDV D/660 and D/OK were used as controls to study the cross-reactivity between clades D/660 and D/OK, which are antigenically different. Monoclonal antibodies generated in our laboratories against D/660 were also used. Cross-reactivity between the two lineages was evaluated by WB and IFA of the mammalian expression system of the HE protein.

#### Western Blot

WB was performed using sucrose-purified viruses and recombinant NP and HE proteins. First, 50 μg of protein was resolved by SDS-PAGE in 7% acrylamide gels and transferred to nitrocellulose membranes. After blocking blots with 5% non-fat dry milk in TBS-Tween 20 (0.1%; TBS-T 20) solution for 2 hr at RT, they were probed with hyperimmune polyclonal antibodies, monoclonal antibodies, and selected bovine antibodies diluted overnight in 5% non-fat dry milk TBS-T 20 were incubated at 4°C. Blots were washed thrice with TBS-T 20 for 10 min at RT and incubated with a goat anti-bovine IgG-HRP (Jackson ImmunoResearch Laboratories, INC, USA) conjugate secondary antibody for bovine sera, a goat anti-rabbit IgG-HRP conjugate secondary antibody for rabbit-polysera, and a goat anti-mouse IgM-HRP conjugate secondary antibody for mAbs for 2 h at RT. Commercial anti-HIS antibody (Invitrogen, Carlsbad, CA) at 1:10,000 was used as primary antibody to confirm expression of the recombinant protein. FBS was used as a negative control. Blots were washed thrice with TBS-T 20 for 10 min, and bands were visualized by staining with 4-chloro-1-naphthol (ThermoFisher, Grand Island, NY).

#### Assay Development and Validation

Positive bovine samples from HI, IFA, and WB were collected to prepare strong positive bovine sera specific for IDV and used for assay development.

#### Indirect ELISA Development and Optimization

Ultraviolet radiation–inactivated, sucrose purified viruses, NP and HE recombinant proteins expressed in *E. coli* were used in indirect ELISA (iELISA). Polysorb microtiter plates (Immunolon Polysorb, 96 well, Thermo Scientific, Waltham, MA) were coated with the appropriate antigen 50 ng/well of ultraviolet radiation–inactivated sucrose-purified whole virus antigen, HE, 100 ng/well; and NP, 25 ng/well (35). Optimal assay conditions (concentration of antigen, serum, anti-bovine biotinylated antibodies, and secondary antibody dilutions) were determined by a checkerboard titration, which gave the highest signal-to-noise ratio. In addition, FBS was used as negative control and a single lot of pooled convalescent IDV serum positive by IFA, HI, and WB was used to establish quality control standards that gave high and low optical density (high OD = 2.0–2.5, low OD = 0.5–1.0, and negative OD = <0.2). The ELISA was performed as previously reported (12, 34).

#### Blocking ELISA Antigen

Using ICV protein as the antigen to generate antibodies, we performed two rounds of purification for the anti-serum: affinity purification using ICV to obtain antibodies that recognize ICV, and perform cross-absorption using IDV to remove the portion of the antibody that could also recognize IDV; hence, the portion left would recognize ICV but not IDV. Blocking ELISA was performed as described previously (34).

#### Blocking Polypeptide ELISA

A combination of equal concentration of each peptide was used as a polypeptide antigen. For optimization, a panel of antibodies specific for ICV and IDV were used to block each alternatively.

#### Fluorescent Focus Neutralization Test

An IDV virus neutralization assay using an FFN format was developed and evaluated using specific, highly neutralized bovine serum samples as described previously (12).

#### Seroconversion of IDV in Experimentally Infected Calves

Calf sera for the study was provided by the Department of Basic Sciences, College of Veterinary Medicine, Mississippi State University, and Mississippi State, Mississippi, USA.

#### Measurement of Statistical Testing Agreement and Correlation

Multiple-comparison inter-rater agreement (kappa measure of association) and Pearson's correlation tests were calculated for all four tests (HI, iELISA [iELISA], FFN, and IFA), using the IBM SPSS version 20 software (SPSS Inc., Chicago, IL).

#### 3D Structure for the ICV, PIC1, PIC2, IDV, PID1, PID2, PID3, and PID4

Are done sing the Phyre2 web portal for protein modeling, prediction and analysis using Kelley LA et al. Nature Protocols 10, 845-858 (2015). In intensive mode it was able to generate a theoretical model covering this region. The images were generated in Pymol (The PyMOL Molecular Graphics System, Version 2.3.5 Schrödinger, LLC).

#### Alignment Between the Selective IDV Peptides Represented in USA Selected Strain and the Non-american IDV Strains

Multiple-sequence alignment of the influenza D virus HEF protein including the four different peptides in comparison to ICV. The HEF protein (NCBI Reference Sequence: NC_036618.1) used in this study were blasted using the NCBI Influenza Virus Resource and the sequences with 97% or more were aligned using muscle v3.8.31, and the aligned sequences of representative viruses at each peptide site are shown along with the consensus sequence (**Figure 4**, Supplementary Materials 1, 3). Alignment between the NP protein of the two clades of IDV to the non-American strains are shown in Supplementary Material 2.

## Results

### HI Assay

All randomly collected serum samples from different states were tested by HI, using D/660 after RDE treatment. About 317 samples were positive from 400 cows and bulls, and 100 serum samples were negative from 7- to 8-month old young cattle and calves. However, the HI test using D/OK showed only 166 positive cases from 400 cows and bulls and 100 serum samples negative out of 100. Remaining samples with known HI titers were from St. Jude Children's Research Hospital.

### Expression of Recombinant IDV-NP and IDV-HE Antigens by the Bacterial System

**Figure 1:**
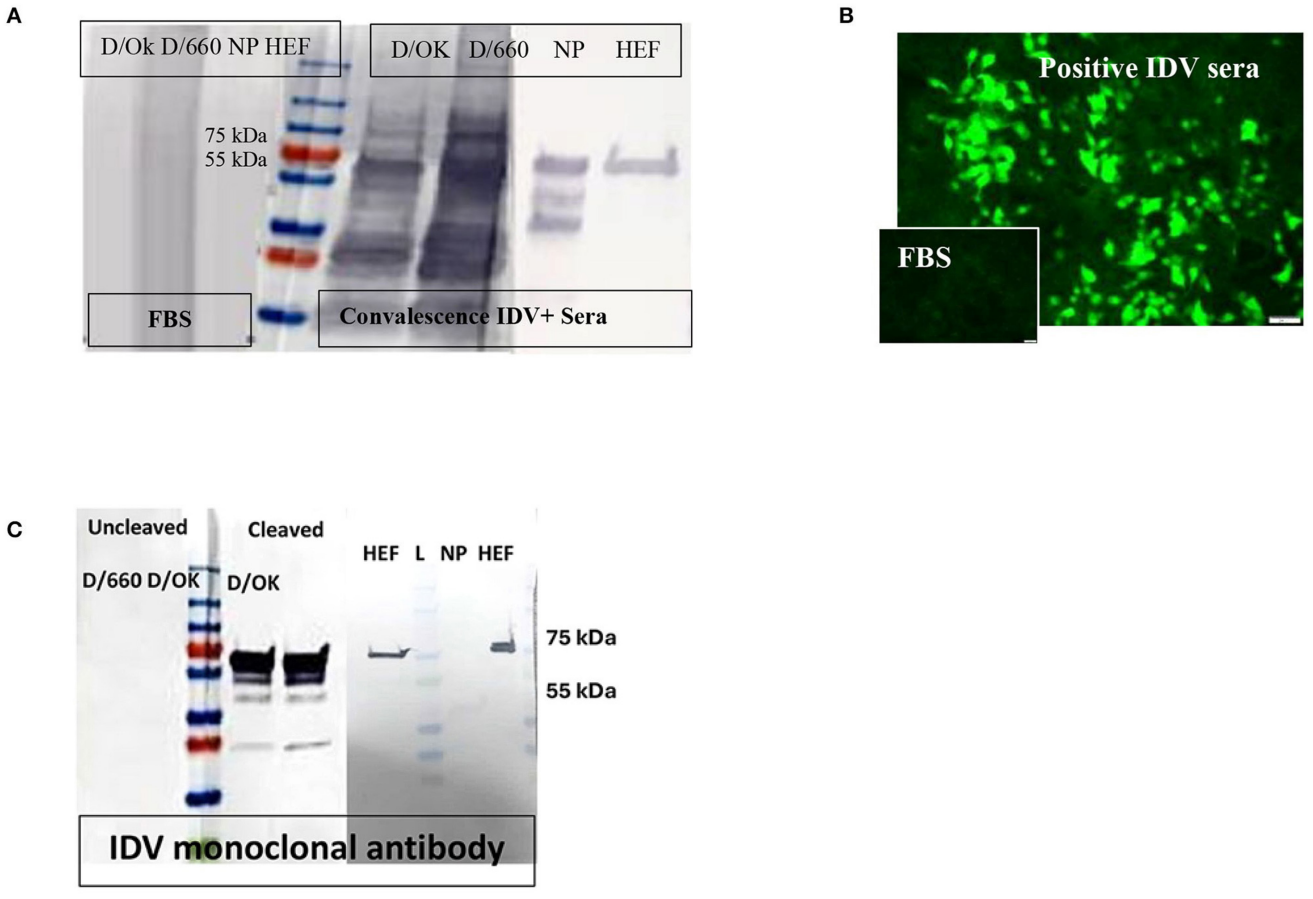
Specificity of recombinant proteins vs. that for the whole virus at the IDV convalescent stage. **(A)** Specificity of recombinant proteins vs. that for the whole virus at the IDV convalescent stage. **(B)** IFA of IDV-infected MDCK cells using positive and negative bovine serum samples and anti-bovine fluorescein isothiocyanate (FITC) including positive convalescent bovine anti-IDV sera showing strong fluorescent staining of virus-infected cells and Negative serum sample showing no specific fluorescent staining. **(C)** Mapping of the IDV mAb.

Protein yield by the IPTG-induced *E. coli* culture was approximately 25 mg IDV-NP/liter of 2XYT medium, with a purity of >95%. Purity of the recombinant protein was assessed by SDS-PAGE, and the expected band of 64 kDa for NP and 65 kDa for HE migrated in Coomassie brilliant blue staining. Specificity of recombinant proteins was confirmed by WB, using convalescent IDV-positive serum, FBS as negative control, and anti-His mAb and anti-rabbit-IDV polysera (Figure 1A).

### Immunofluorescence Assay

About 166 of 400 field samples tested by the IFA were classified as positive and 100 of 100 as negative. The IDV-positive control serum showed clear cytoplasmic fluorescence starting at a 1:40 dilution, whereas the IDV-negative serum did not (Figure 1B). We developed an accurate and sensitive IFA specific for both lineages of IDV (Figure 1B) by using control-positive and control-negative serum samples evaluated by WB to avoid non-specific binding (Figure 1A).

### Western Blot

WB was done for three purposes. First, WB was used to investigate some selective serum samples against the whole virus, NP, and HE, using the anti-bovine HRP antibody to confirm seroconversion status (Figure 1A). Second, WB was used to map the monoclonal antibody generated in our laboratory. To map the mAb of isotype IgM, sucrose-purified whole virus D/660 and D/OK were treated with trypsin for 1 h at a concentration of 10 μg/ml before running the gel. Non-treated viruses were used as a control along with NP and HE proteins. Our mAb cross-reacted with the HE recombinant protein and did not cross-react with NP. It also cross-reacted with both D/660 and D/OK under trypsin treatment and did not react with the whole virus (Figure 1C) compared to non-cleaved virus with anti-rabbit sera. To map whether IDV produced mAbs that could be used in competitive ELISA, we used WB. We blotted the whole virus of both IDV clades in two forms: cleaved or not cleaved by trypsin. Our IDV-mAb did not recognize the non-cleaved whole virus but reacted strongly with the cleaved version (Figure 1C). Blotting this mAb with the two recombinant NP and HE bacterial expressed proteins showed no recognition with NP but a strong signal with HEF (Figure 1C).

### Indirect ELISA

**Figure 2:**
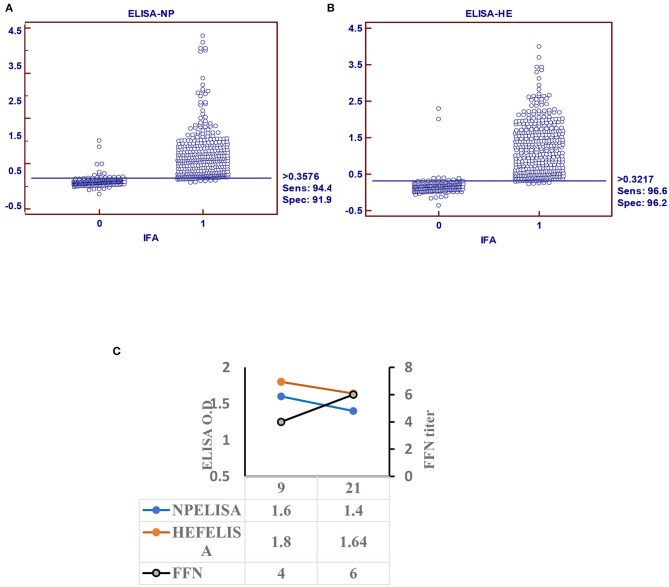
Indirect ELISA specific for IDV (iELISA). **(A)** Receiver operator characteristics (ROC) and diagnostic sensitivity and specificity of IDV-NP. **(B)** ROC analysis and diagnostic sensitivity and specificity of IDV-HE (MedCalc version 11.1.1.0, MedCalc software, Mariakerke, Belgium) In each panel, dot plot on the left and right denote negative and positive testing populations, respectively. The horizontal line bisecting the dot plots denotes the cutoff value for optimal diagnostic sensitivity and specificity. **(C)** Seroconversion of IDV in infected calves using ELISA-based NP, HE, and FFN in calves experimentally infected with IDV.

Receiver operating characteristic analysis of the indirect ELISA with the IFA was used to determine sensitivity and specificity values and cutoffs. Optimal cutoff values and corresponding sensitivity and specificity of NP- and HEF-based tests are shown in Figures 2A,B, respectively. Receiver operating characteristic analysis for the NP-iELISA showed 94.4% sensitivity and 91.9% specificity with a cutoff of 0.357. Receiver operating characteristic analysis for the HEF-iELISA showed an estimated sensitivity and specificity of 96.6 and 96.2%, respectively, with a cutoff of 0.321. We detected 332 positive samples out of 350 expected positive samples and 120 negative samples from 150 archived samples (1984–1999), since IDV did not circulate before 2003 (5). We found an ~90% preliminary prevalence of IDV in dairy cattle and 4–7% prevalence in healthy 7-month-old calves. We optimized and validated the HEF- iELISA, and our results showed high specificity and sensitivity (Figure 2B) as well as high correlation with the two neutralizing tests HI and FFN. These results not only confirm the specificity of HI and FFN directed toward protective antibodies against HEF, but also indicate that neutralizing tissue culture-based assays that require BSL2 facilities and a highly prepared lab to perform ELISA to determine neutralizing antibodies can be easily performed in a traditional lab, clinical lab, and veterinary clinics (Table 1). Based on the blast and alignment analysis, both Np and HEF ElISA developed here can be used for the non-American IDV strains (**Figure 5** and Supplementary Material 2).

**Table 1:** Pearson correlation coefficients for diagnostic assays between HI results after log-2 transformation and FFN.

	**Total no. 450**	**HEF-iELISA**	**NP-iELISA**	**FFN (Log)**	**HI Log**	**IFA Log**
ELISA-HE	Correlation coefficient		0.517	0.548	0.522	0.556
	Significance level P		<0.0001	<0.0001	<0.0001	<0.0001
ELISA-NP	Correlation coefficient	0.517		0.484	0.435	0.512
	Significance level P	<0.0001		<0.0001	<0.0001	<0.0001
FFN (Log)	Correlation coefficient	0.548	0.484		0.581	0.705
	Significance level P	<0.0001	<0.0001		<0.0001	<0.0001
Hi_Log	Correlation coefficient	0.522	0.435	0.581		0.732
	Significance level P	<0.0001	<0.0001	<0.0001		<0.0001
IFA_log	Correlation coefficient	0.556	0.512	0.705	0.732	
	Significance level P	<0.0001	<0.0001	<0.0001	<0.0001	

*Correlation coefficient values were 0.581 between HI and FFN, 0.548 between HI and HEF-iELISA, and 0.522 between iELISA based on HEF and FFN-log2*.

### Seroconversion of Influenza D Virus in Experimentally Infected Calves

Calf sera showed seropositivity at day 9 post infection with Np and HEF-iELISA, with high titers of neutralization seen with FFN at 21 DPI (Figure 2C).

### FFN

The FFN assay was initially run by using well-recognized positive convalescent serum samples by WB, IFA, HI, and ELISA. Essentially, the 100 negative samples showed serum FFN endpoint titers of <1:20 and included calves 6–7 months old. Of the 350 positive sample set of cows and bulls, 349 had endpoint titers from 1:80 to 1:1280. We optimized and developed FFN by using high-throughput methods specific for IDVs.

### Statistical Correlation Among HI, FFN, and HEF-iELISA

Pearson's correlation test among all diagnostic platforms showed high correlation values among assays. There was a good correlation between HI results after transformation to log 2: FFN value was 0.732 between HEF-iELISA and FFN-log 2 was 0.556, and that of iELISA based on HEF and HI log 2 was 0.532 (Table 1). The HI results using both clades D/660 and D/OK were different in seropositive status but the FFN results were able to cover both. Our IFA, iELISAs and FFN tests specific for IDV will detect both the protective and non-protective antibodies against the two IDV antigenically different clades that co-circulating in the U.S. Indirect HE ELISA and FNN showed a high correlation with HI results, which confirm that the HI test can be replaced by HEF- iELISA and FFN. The HI results from clades D/660 and D/OK revealed differences in seropositive status, but FFN results were consistent.

### Cross-Reactivity Between the Two Lineages of IDV

**Figure 3:**
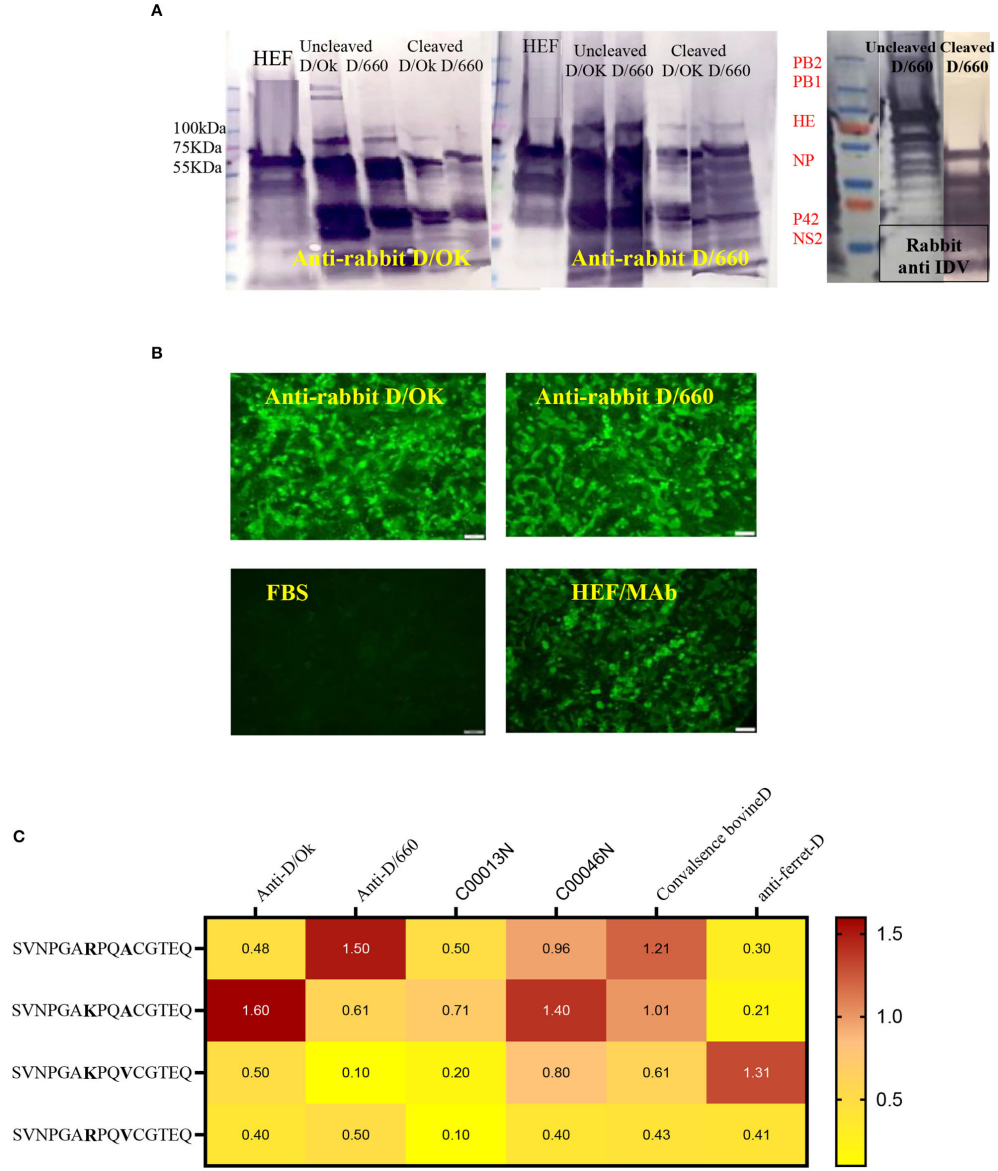
Cross-reactivity between two IDV different clades of D/660 and C/OK. **(A)** Cross-reactivity between the two IDV different clades of D/660 and C/OK using specific polyclonal rabbit anti-IDV sera against each clade and using polyclonal rabbit anti-IDV/D/660 and polyclonal rabbit anti-IDV/C/OK sera against cleaved and non-cleaved viruses. **(B)** Cross reactivity of the mutant HE protein at the site 212 amino acid. MDCK cells were stained with both IDV lineages of anti-rabbit-sera and mAb after transfection in MDCK cells. **(C)** List of peptides used and recognition of both D/OK and D/660 antibodies.

Cross-reactivity between IDV-D/660 and IDV-D/OK was determined using rabbit antisera specific for both IDV lineages generated in our laboratories. We used WB with different epitopes from whole-virus D/660, D/OK, trypsin and no trypsin treated D/660, trypsin and no trypsin treated D/OK, and recombinant N and HE proteins against rabbit antisera specific for both lineages alternatively. Figure 3A shows that both anti-rabbit sera reacted with both viruses treated or not treated with trypsin, demonstrating cross-reactivity between the two clades when using our rabbit antisera. Therefore, our rabbit antisera will serve as an accurate tool for general diagnosis of IDV in the US. Moreover, to investigate whether the amino acid 212, located in the apex of the HE receptor binding domain, has a critical role in antibody recognition, we introduced a deletion mutation in this region and cloned the mutant in the PcDNA3.1 mammalian expression vector and then transfected MDCK cells with the construct. Transfected cells were stained by both rabbit polyclonal antisera specific to the two IDV clades and cross-reacted with mutant HEF. The mutation introduced by deleting the amino acid site 212 of the HE protein and transfection in MDCK cells using lipofectamine 3000 did not affect the antigenic recognition of the epitope against mAb and rabbit antisera for both lineages using IFA (Figure 3B).

### Peptide ELISA to Assess the Heterogeneity of Recognition Between the Two Lineages

Despite the genetic differences between the two lineages, mutation in position 212 did not show differences in the recognition of antibodies. Therefore, we investigated the importance of K, R, A, and V amino acids in the heterogeneity of the IDV-circulated lineage and found that positions 215 play an important role in heterogeneity (Figure 3). We treated both sucrose-purified viruses with TPCK-treated trypsin to cleave the HE protein and used both rabbit polyclonal antisera against both clades alternatively (Figure 3A). Both cleaved and non-cleaved viruses reacted to both rabbit antisera. Also, both polyclonal antisera specific to both clades reacted with the D/OK HE recombinant protein. Therefore, we developed peptide ELISA using a panel of four different peptides. Each peptide had 14 amino acids covering the region from positions 206 to 220 (Figure 3C). Both positions 212 and 215 are important for the heterogeneity between the two lineages (Figure 3, graphical abstract and Table 2). Peptide ELISA with the anti-IDV ferret serum was positive with D/OK and D/660 m in position 215 (A to V), with no cross reaction with D/660. The list of peptides and recognition of both D/OK and D/660 antibodies are given in (Figure 3C and Table 2). These results highlight the importance of position 215 with 212 in the zoonotic importance of IDV. Using the peptides ELISA with anti-IDV from ferret showed only positive with the D/OK lineage and D/660 mutant in position 215 (A to V).

**Table 2:** Summary for the representative assays in this study compared to the shaded standard assay used for influenza viruses.

**Epitope**	**Sequence**	**Assay**	**Analyte**	**BSL2**	**Sample/host**	**Application**	**Limitation**
IDV/OK/or IDV/660	Whole virus	FFN	IgG/IgM	Yes	Bovine serum	IDV antibodies	BSL2/expensive
IDV/OK/or IDV/660	Whole virus	IFA	Poly	Yes	Bovine serum	IDV antibodies	BSL2/expensive
IDV-NP	40-600 AA	Indirect ELISA	IgG	No	Bovine serum	IDV antibodies	Very specific
IDV-HEF	60-400 AA	Indirect ELISA	IgG	No	Bovine serum	IDV antibodies	Very specific
IDV/OK/Whole-Viral-Ag	Sucrose purified virus	Indirect ELISA	IgG	No	Bovine serum	IDV/OK (swine clade) antibodies	Very specific
IDV/660/Whole-Viral-Ag	Sucrose purified virus	Indirect ELISA	IgG	No	Bovine serum	IDV/660 (Bovine clade) antibodies	Very specific
IDV peptide	SVNPGA**R**PQ**A**CGTEQ (206-220aa)	Indirect ELISA	IgG	No	Bovine serum	IDV/660 (Bovine clade) antibodies	Not cheap
IDV peptide	SVNPGA**K**PQ**A**CGTEQ (206-220aa)	Indirect ELISA	IgG	No	Bovine serum	IDV/OK (swine clade) antibodies	Not cheap
IDV peptide	SVNPGA**R**PQ**V**CGTEQ (206-220aa)	Indirect ELISA	IgG	No	Bovine serum	IDV/660 (Bovine clade) antibodies	Not cheap
IDV peptide	SVNPGA**K**PQ**V**CGTEQ (206-220aa)	Indirect ELISA	IgG	No	Bovine serum	IDV/OK (swine clade) antibodies	Not cheap
HICV/Whole-Viral-Ag	Sucrose purified virus	Indirect ELISA	IgG	No	Human serum	HICV antibodies	Very specific
IDV/OK/Whole-Viral-Ag	Sucrose purified virus	Blocking ELISA	IgG	No	Human serum	IDV antibodies	Very specific
HICV/Whole-Viral-Ag	Sucrose purified virus	Blocking ELISA	IgG	No	Human serum	HICV antibodies	Very specific
Peptide 1 IDV (PID1)	AESSVNPGAKPQV (203–215 aa)	Indirect ELISA	IgG	No	Human serum	IDV antibodies	Not cheap
Peptide 2 IDV (PID2)	IANAGVK (286–292 aa)	Indirect ELISA	IgG	No	Human serum	IDV antibodies	Not cheap
Peptide 3 IDV (PID3)	KTDSGR (423–428 aa)	Indirect ELISA	IgG	No	Human serum	IDV antibodies	Not cheap
Peptide 4 IDV (PID4)	RTLTPAT (448–455 aa)	Indirect ELISA	IgG	No	Human serum	IDV antibodies	Not cheap
Peptide 1 ICV (PIC1)	RTDKSNSAFPRSAD (74–87 aa)	Indirect ELISA	IgG	No	Human serum	HICV antibodies	Not cheap
Peptide 2 ICV (PIC2)	GSRKESGGGVTKES (484–497 aa)	Indirect ELISA	IgG	No	Human serum	HICV antibodies	Not cheap
IDV-Polypeptide	Equal concentration of P1D1 to PID4	Blocking ELISA	IgG	No	Human serum	IDV antibodies	Not cheap
ICV-Polypeptide	Equal concentration of (P1D1-2)	Blocking ELISA	IgG	No	Human serum	HICV antibodies	Not cheap
HI test	IDV/OK, IDV/660, HICV	HI	Poly	Yes	Human/bovine	Based on the virus	Time/less sensitivity
Microneutralization assay	IDV/OK, IDV/660, HICV	ELISA based tissue culture immunoperoxidase	poly	Yes	Human/bovine	Based on the virus	Time consumed
Reverse-transcription PCR	IDV/OK, IDV/660	PCR	Nucleic acid	Yes	Swap	Number of cycles	Expensive

**Table 3:** IDV similarities to ICV IDV shares 50% amino acid sequence identity with ICV, lacks the neuraminidase gene, and has seven genes instead of eight seen in IAV and IBV (4).

**IDV gene**	**Similarities to ICV**
PB2	58.5%
PB1	66.7%
P3	57.29%
HEF	55.7%
NP	51.4%
NS	47.5%
P42	52.4%

*Sequence similarity between ICV and IDV is as follows: PB2 gene, 58.5%; PB1 gene, 66.7%; P3 gene, 57.29%; HEF gene, 55.7%; NP gene, 51.4%; NS gene, 47.5%; and P42 gene, 52.4%*.

#### Structural Representation of the Haemagglutinin Esterase Fusion Glycoprotein From the Influenza C Virus and the Influenza D Virus

**Figure 4:**
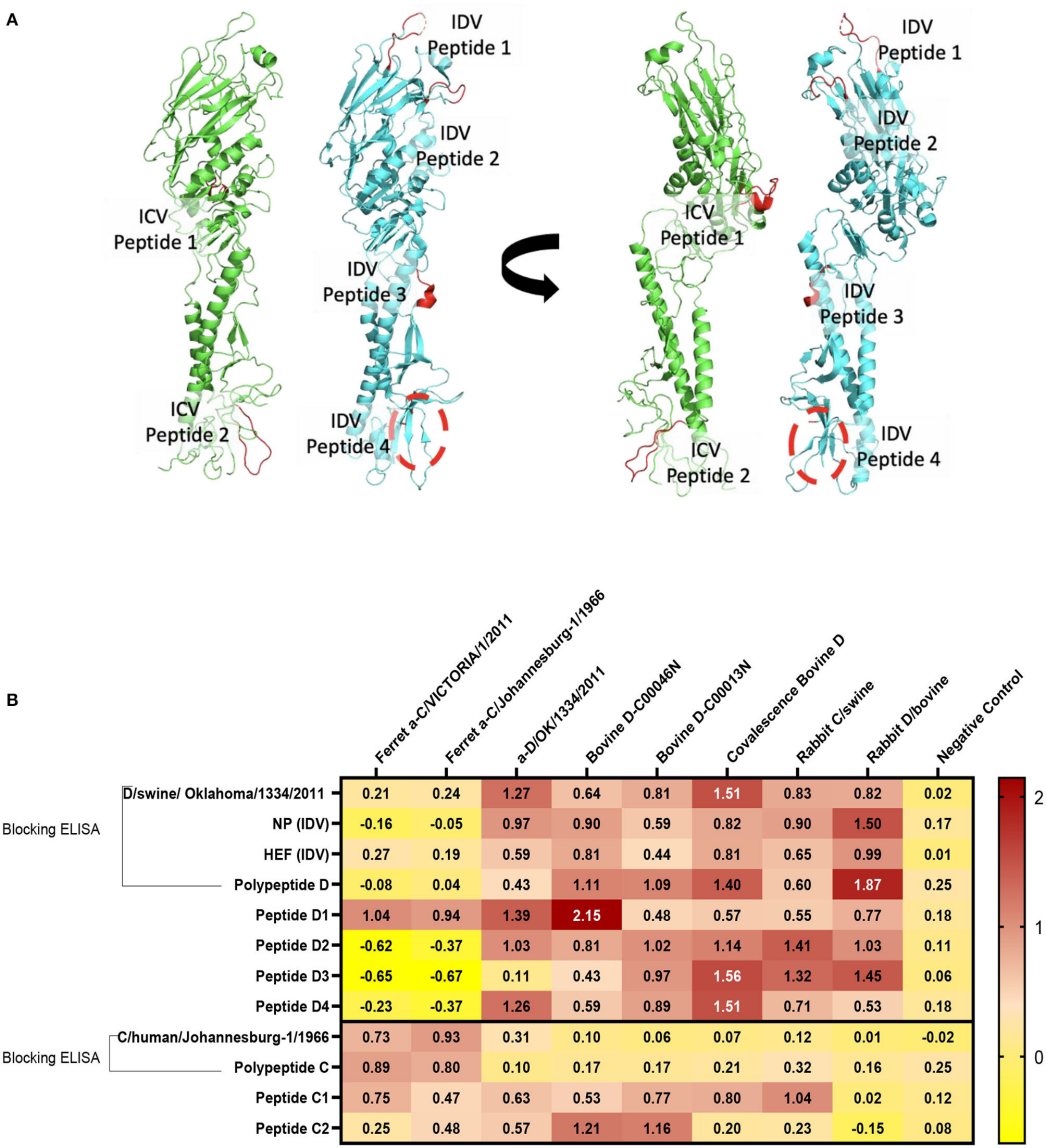
Differentiation between IDV and ICV. **(A)** Structural representation of the haemagglutinin esterase fusion glycoprotein from the influenza C virus in green (PDB:1FLC) and the influenza D virus in blue (PDB:5E64). Peptides of interest are represented in red. ICV peptide one is found in esterase domain one and has the sequence RTDKSNSAFPRSAD. ICV peptide two is found in fusion domain two and has the sequence GSRKESGGGVTKES. IDV peptide one is found in the receptor domain and has the sequence AESSVNPGAKPQVCGT. IDV peptide two is also found in the receptor domain and has the sequence IANAGVK. IDV peptide three is found in the fusion domain and has the sequence KTDSGR. An enzymatic cleavage site was noted adjacent to IDV peptide three. IDV peptide four falls within a 17 amino acid region not represented in structural data. Its location is highlighted with a dashed circle. IDV peptide four has the sequence RTLTPAT and occupies the fusion domain. **(B)** NP, HEF, and IDV antigens were blocked by anti-ICV antibodies. ICV was blocked by anti-IDV antibodies. Compared to blocking ELISA, polypeptides and single peptide ELISA had higher sensitivity and specificity of peptides to differentiate between IDV and ICV.

Structural representation of the haemagglutinin esterase fusion glycoprotein from the influenza C virus in green [PDB:1FLC (32, 36)] and the influenza D virus in blue [PDB:5E64 (32)]. ICV peptide one (PIC1) is found in esterase domain one and has the sequence RTDKSNSAFPRSAD. ICV peptide two (PIC2) is found in fusion domain two and has the sequence GSRKESGGGVTKES (Figure 4A). IDV peptide one (PID1) is found in the receptor domain and has the sequence AESSVNPGAKPQVCGT. IDV peptide two (PID2) is also found in the receptor domain and has the sequence IANAGVK. IDV peptide three (PID3) is found in the fusion domain and has the sequence KTDSGR. An enzymatic cleavage site was noted adjacent to IDV peptide three. IDV peptide four (PID4) falls within a 17 amino acid region not represented in structural data. Its location is highlighted with a dashed circle. IDV peptide four has the sequence RTLTPAT and occupies the fusion domain (Figure 4A).

#### Blocking Polypeptide ELISA and Single-Peptide ELISA to Differentiate Between ICV and IDV

On the basis of the alignment between the HEF of ICV and IDV, we selected four different conserved peptides for each virus that have different amino acid sequences. The amino acid sequences had seven to 16 amino acids. A combination of equal concentration of each peptide was used as a polypeptide antigen. For optimization, a panel of antibodies specific for ICV and IDV were used (Figure 4B). To establish a serological test to differentiate between two closely related viruses, we developed peptides, blocking polypeptides, and blocking whole antigen ELISAs. Our IDV blocking and peptide 2–4 ELISAs can be used to detect and distinguish between ICV and IDV. IDV was more conserved and had three different peptides that did not cross-react with ICV, whereas the only two conserved ICV peptides were positive for IDV (Figure 4B). This indicates that ICV can cross-react with IDV and vice versa.

#### Representation of the IDV HEF Protein and Peptides Among Non-american IVD

**Figure 5:**
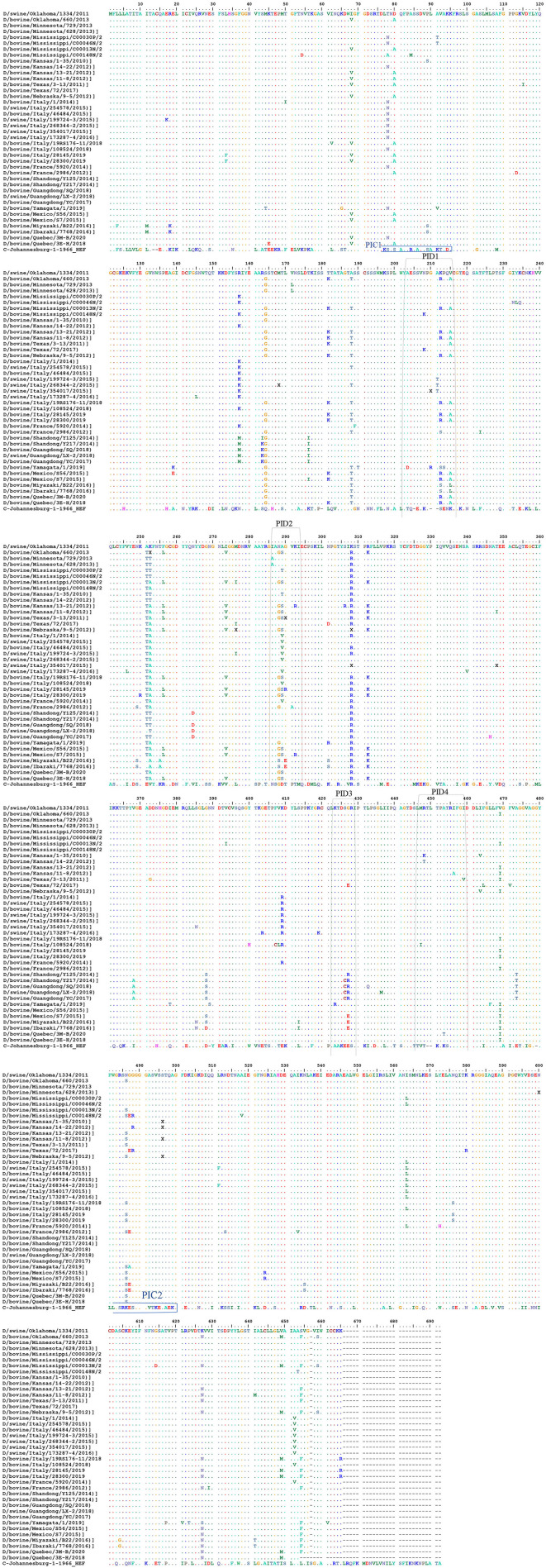
Alignment between the selective IDV peptides represented in USA and the non-American IDV strains. Representation of the IDV HEF protein including the four IDV peptides of the strain in our study. Multiple-sequence alignment of the influenza D virus HEF protein and four different peptides in comparison to ICV. The HEF protein used in this study were blasted using the NCBI Influenza Virus Resource and the sequences with 97% or more were aligned using muscle v3.8.31, and the aligned sequences of representative viruses at each peptide site are shown along with the consensus sequence. Dots represent identical residues, PID1, PID2, PID3, and PID4 are outlined, and the location of the fusion peptide is shown.

Representation of the IDV HEF protein including the four IDV peptides of the strain in our study. The IDV Peptides showed more similarities to the European strains than of the Asian. The two clades IDV virus led sequence are cataloged with other virus sequences from the blast then an alignment using Bio edit program has been done. As part of this process, we compared the ICV sequence with the other virus sequences and looks for differences among them to visually represent how genetically different the Peptides are from each virus strain to other (Figure 5 and Supplementary Materials 1, 3). PID1 showed a 100% similar to (D/bovine/Guangdong/SQ/2018), (D/swine/Guangdong/LX-2/2018), (D/bovine/Guangdong/YC/2017), (D/bovine/Shandong/Y125/2014), (D/bovine/Shandong/Y217/2014), (D/bovine/Guangdong/SQ/2018) (D/bovine/France/5920/2014), (D/bovine/Italy/108524/2018), (D/swine/Italy/173287-4/2016), (D/swine/Italy/268344-2/2015), (D/bovine/Italy/46484/2015) and (D/swine/Italy/254578/2015) (Figure 5 and Supplementary Material 3), while PID2 showed 100 % similarities to (D/bovine/Guangdong/SQ/2018), (D/swine/Guangdong/LX-2/2018), (D/bovine/Guangdong/YC/2017), (D/bovine/Shandong/Y125/2014), (D/bovine/Shandong/Y217/2014), (D/bovine/Guangdong/SQ/2018), and (D/swine/Italy/354017/2015) (Figure 5 and Supplementary Material 3). PID3 showed 100 % similarities with (D/bovine/Quebec/3E-H/2018), (D/bovine/Quebec/3M-B/2020), (D/bovine/Mexico/S56/2015), (D/bovine/France/5920/2014), (D/bovine/France/2986/2012), (D/bovine/Italy/108524/2018), (D/swine/Italy/173287-4/2016), (D/bovine/Italy/46484/2015), (D/swine/Italy/254578/2015), (D/swine/Italy/354017/2015), (D/bovine/Italy/28300/2019), (D/bovine/Italy/28145/2019), (D/bovine/Italy/19RS176-11/2018), (D/swine/Italy/199724-3/2015), and (D/bovine/Italy/1/2014), while it showed 90% to (D/bovine/Yamagata/1/2019).

PID 4 showed 100% similarities to all the European and Eurasian strains except a 90 % to (D/bovine/Italy/108524/2018). (Figure 5 and Supplementary Materials 1, 3).

## Discussion

Although the HI test is commonly used for IVs, it cannot be used to detect the antibodies against the two genetic lineages IDV, D/OK-like virus (swine clade) and D/660-like virus (bovine clade), which are antigenically distinct (37). Therefore, each lineage requires a specific standard test. For better diagnosis of IDV, our aim was to develop general, sensitive antibody-based tissue culture assays that can detect both lineages of IDV. Neutralization assays (e.g., MN) cannot detect cross-reactive antibodies in the highly conserved stem region of different IVs, especially in the elderly (38). Also, they identify protective antibodies against specific epitopes, but not non-neutralizing antibodies produced by the immune system against other epitopes (e.g., PB1, PB2, M, and other IV proteins), which can reflect the serostatus of the animal but are missing in MN or HI tests. There are different formats of the MN assay worldwide, such as a 2-day ELISA protocol (39), a 3-day HA protocol, and a 7-day HA protocol (18). Because time is critical for sero-epidemiology and early diagnosis, an accurate and shorter protocol of 2 days is optimal (12).

Our proposed diagnostic assays can be a sensitive and accurate diagnostic tool for high-throughput surveillance and detection of both D/OK-like and D/660-like IDVs co-circulating in the same herds in both research and clinical labs. We needed IFA and FFN for tissue culture–based analysis for analyzing virus interactions. The IFA is the gold standard of infectious disease serology, rapid, accurate, highly sensitive and highly specific technique that can be used for viral identification (40, 41). IFA has been used efficiently for the management and surveillance of several IVs (42, 43). However, the IFA assay is very expensive to use in surveillance and cannot differentiate between neutralizing and non-neutralizing antibodies (12). FFN is a neutralization test based on high avidity binding of rabbit polyclonal antisera prepared in our lab against IDV as a detection antibody. The FFN assay can detect neutralizing antibodies against both IDV clusters, using either type of IDV as the virus indicator. The biggest advantage of FFN over iELISA is that iELISA requires species-specific enzyme-conjugated antibodies but FFN does not (34). Furthermore, FFN detects only neutralizing antibodies (35), so it can be used for further optimization by using milk and colostrum samples from cow herds that experienced an acute IDV outbreak. Serum samples can be used to quantify maternal and acquired immunity transferred to calves (44). Unfortunately, IFA and FFN require BSL2 facilities that are not available in many veterinary centers. Hence, our second aim was to develop an iELISA using specific epitopes to detect two lineages of IDVs simultaneously.

Epitope-iELISA accurately detects neutralizing and non-neutralizing antibodies against the IDV in non-BSL2 laboratories and veterinary clinics (12) and is cost-effective and sensitive (12, 22). The IDV NP is considered a genus-specific antigen that can distinguish among different genera of IVs. NP is responsible for the loss of cross-recognition of viral antigens between different IVs (4). NP is a conserved domain with 94% similarities between IDV swine and bovine clades (Supplementary Material 2). Therefore, it represents the best epitope target for general seroprevalence of IDV in clinical labs and veterinary clinics. We established two separate and optimized iELISAs based on NP and HEF. The HEF epitope is specific for ICV and IDV but not IAV and IBV (32). The sensitivity and specificity of our NP- iELISA were very good (Figure 2A), However, the specificity was higher than the sensitivity, which may be related to fewer negative samples than positive samples in our study. Therefore, further analysis of known negative samples are next steps in optimizing the test. NP-iELISA can detect antibodies that target common viral proteins (34) and can be effective for large-scale surveillance of IDV in herds. The HEF protein of IDV recognizes and binds the specific receptor, and consequently mediates viral entrance and virus infection (23). Our HEF- iELISA showed high sensitivity and specificity (Figure 2B). Furthermore, comparison of seroprevalence of 6- to 7-month-old calves and adult cattle revealed a significant increase of 10% in 8-month-old calves compared to 89% in adult cattle (8). Our results highlight the high spread rate of IDV among cattle in the US, in contrast to a study reporting a spread rate of 11.3% in calves and 66.5% in adult cows (9). The variation seen between calves and adult cows could be due to passive immunity against IDV transferred from positive adult cattle to calves, which may aid temporary protection (9). Calf sera showed seropositivity at 9 days post infection and high titers of neutralization by FFN at 21 days post infection (Figure 2C).

The above assays can determine whether the status of tested samples is positive or negative against IDV in general but cannot determine the specific lineage (graphical abstract). Therefore, our third aim was to develop another diagnostic assay to differentiate between the two IDV lineages. The IDV/D/660 clade has a K replacing R of the D/OK clade at position 212. HI results and molecular models of the two clades revealed that amino acid 212 plays an important role in IDV antigenicity and antibody recognition (37). For understanding the two IDVs lineages at the molecular level, we developed an assay to serologically differentiate between the two viral clades. First, we tested cross-reactivity between the two clades using our rabbit polyclonal antisera against both D/OK and D/660. A strong cross-reactivity was found when using the alternative rabbit polyclonal antisera (Figures 3A,B). A deletional mutation at the amino acid site 212 in only the HEF protein did not affect antigenic recognition of the epitope against mAb and rabbit antisera for both lineages (Figure 3B), which suggested that the amino acid 212 region alone does not cause a difference in HI titers between the two clades. Another region such as V115I or D372G in the esterase domain (37) may also play a critical role in antigenic differences in recognizing the receptor.

Peptide ELISA with anti-IDV antibodies from ferret were only positive with the synthetized peptide with a mutation in position 215 (A to V) in both the swine lineage D/OK and the bovine lineage D/660 (Figure 3C). This indicates that IDV is a zoonotic hazard, and more samples of individual coworkers handling swine and bovine lineages need to be screened. Our results from peptide ELISA can be an important tool to differentiate between the zoonotic ability of both lineages and determine drifts in HEF protein between swine and bovine lineages after transmission.

To date, viral zoonotic diseases maintain to cause pandemic in human (45). Although the zoonotic potential of IDV remains unclear, it can efficiently replicate and transmit in ferrets which considered a good model for human IVs (46). In contrast to a recent study in Scotland showing that no evidence of seroprevalence of IDV but a seroprevalence of ICV in human respiratory samples (47), another study revealed high seroprevalence of IDV in sera from humans exposed to calves, indicating the zoonotic potential of IDV (30). Importantly, the frequent mutations in IVs necessitate measures to prepare for the potential threat to human and public health. Therefore, it is critical to develop diagnostic tools and assays to differentiate between ICV and IDV due to their similar genomic structures. Such accurate assays are required for serological and epidemiological analysis to clarify the complexity and evolution and eliminate misdiagnosis between ICV and IDV. Here, we developed a new diagnostic and serological-based assay, which will serve as an excellent tool for veterinarians and researchers in IDV surveillance and vaccine evaluation, along with diagnostic assays to serologically differentiate between ICV and IDV (Figure 4 and Table 2). Our results agree with those from a previous study showing that IDV clusters most closely with and is derived from human ICV (48), which indicates that ICV can cross-react with IDV and vice versa (Figure 4B). Blocking ELISAs and IDV peptides IANAGVK (286–292 aa), KTDSGR (423–428 aa), and RTLTPAT (448–455 aa). Our blocking ELISA, PID3 and PID4 will be an excellent approach to differentiate between IDV and ICV in human samples in USA and Europe based on the alignment and blast results from the NCBI and Uniport (Figure 5, Supplementary Materials 1, 3).

In conclusion, our study and the assays we have developed will play key roles in seroprevalence studies, surveillance, diagnosis of IDV, and vaccine evaluation (graphical abstract and Table 2). Also, our new diagnostic tests will play a critical role in vaccine evaluation. The indirect HE ELISA and FNN showed the possible replacement of the HI test by HE based iELISA and FFN. Our IDV tissue culture–based assays IFA and FFN are important for research labs and vaccine evaluation, and our NP-iELISA and/or HEF-iELISA can be used in clinical laboratories lacking BSL2 facilities. Peptide-iELISA of the two IDV lineages highlighted the importance of position 215 with 212 in the zoonotic importance of IDV and the heterogeneity between the two lineages. Blocking ELISAs and IDV peptides IANAGVK (286-292aa), KTDSGR (423-428aa), and RTLTPAT (448-455aa) will be a great tool to differentiate between IDV and ICV in human samples. The two IDV's lineage peptides-iELISA highlighted the importance of position 215 with 212 in the zoonotic importance of IDV and the heterogeneity between the two lineages.

## Data Availability Statement

The raw data supporting the conclusions of this article will be made available by the authors, without undue reservation.

## Author Contributions

FO: conceptualization, writing, and visualization. AS and FO: methodology, software, validation, and formal analysis. EN and RW: investigation, supervision, project administration, and funding acquisition. RW: resources. FO and RW: data curation. AS and FO: writing, review, and editing. All authors contributed to the article and approved the submitted version.

## Conflict of Interest

The authors declare that the research was conducted in the absence of any commercial or financial relationships that could be construed as a potential conflict of interest.
