# Correction: New diagnostic assays for differential diagnosis between the two distinct lineages of bovine influenza D viruses and human influenza C viruses

**DOI:** 10.3389/fvets.2025.1654802

**Published:** 2025-08-19

**Authors:** Faten A. Okda, Elizabeth Griffith, Ahmed Sakr, Eric Nelson, Richard Webby

**Affiliations:** ^1^Department of Infectious Diseases, St. Jude Children's Research Hospital, Memphis, TN, United States; ^2^Veterinary Division, National Research Center, Cairo, Egypt; ^3^Department of Chemical and Therapeutic, St. Jude Children's Research Hospital, Memphis, TN, United States; ^4^Department of Business Administration and Management, Dakota State University, Madison, SD, United States; ^5^Veterinary & Biomedical Sciences Department, Animal Disease Research and Diagnostic Laboratory, South Dakota State University, Brookings, SD, United States

**Keywords:** influenza D viruses, influenza C viruses, differential diagnosis, peptide ELISAs, blocking ELISA, diagnostic assay

There was a mistake in Figure 1C, as published. In the published figure, one line was missing. The corrected Figure 1 and its caption appears below.

**Figure 1:**
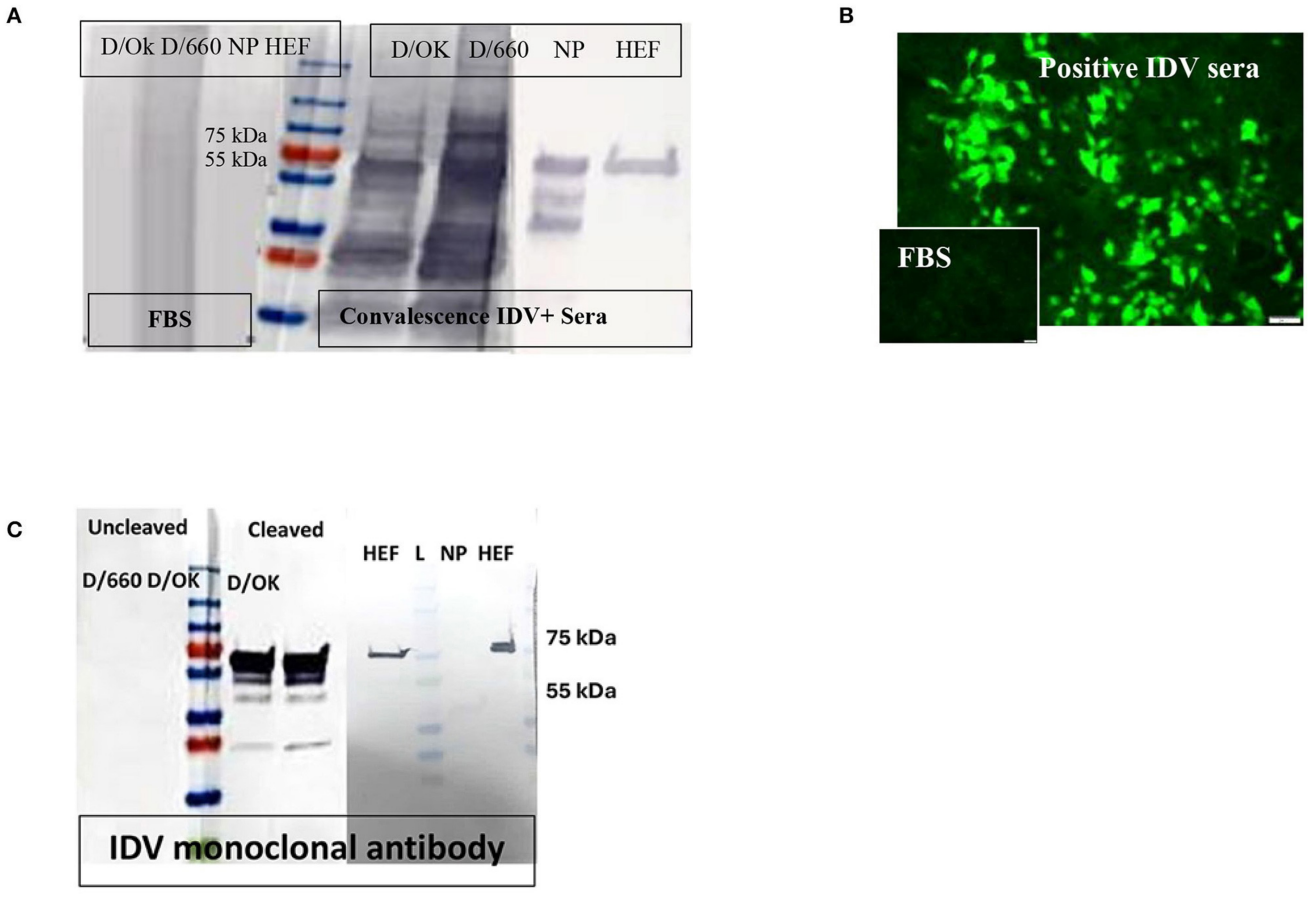
Specificity of recombinant proteins vs. that for the whole virus at the IDV convalescent stage. **(A)** Specificity of recombinant proteins vs. that for the whole virus at the IDV convalescent stage. **(B)** IFA of IDV-infected MDCK cells using positive and negative bovine serum samples and anti-bovine fluorescein isothiocyanate (FITC) including positive convalescent bovine anti-IDV sera showing strong fluorescent staining of virus-infected cells and Negative serum sample showing no specific fluorescent staining. **(C)** Mapping of the IDV mAb.

In the published article, Supplementary Images 1–5 were omitted. The files have now been published.

The original version of this article has been updated.

