# Supplementary material for: New Diagnostic Assays for Differential Diagnosis Between the Two Distinct Lineages of Bovine Influenza D Viruses and Human Influenza C Viruses
Source: Front Vet Sci. 2020 Dec 11;7:605704. doi: 10.3389/fvets.2020.605704 (PMC7759653; doi:10.3389/fvets.2020.605704)
Supplement: Supplementary file 3 [file Data_Sheet_3.PDF]

```
                                   10         20         30         40         50         60         70         80         90         100
                           ....|....| ....|....| ....|....| ....|....| ....|....| ....|....| ....|....| ....|....| ....|....| ....|....|
YP_009449559HEF(D/swine/Oklaho MFLLLATITA ITACQAEREL ICIVQRVNES FSLHSGFGGN VYSMKTEPMT GFTNVTKGAS VINQKDWIGF GDSRTDLTND QFPASSDVPL AVAKKFRSLS
AGS48804HEF(D/bovine/Oklahoma/ .......... .......... .......... .......... .......... .......... .......V.. .........A .......... ..........
AGS48797HEF(D/bovine/Minnesota .......... .......... .......... .......... .......... .......... .......... .......... .......... ..........
AGS48812.HEF(D/bovine/Minnesot .......... .......... .......... .......... .......... .......... .......... .......... .......... ..........
ALE66342(D/bovine/Mississippi/ .......... .......... .......... .......... .......... .......... .......... .......N.. .......... T.........
ALE66335(D/bovine/Mississippi/ .......... .......... .......... .......... .......... .......... .......... .......N.. .......... T.........
ALE66340HEF(D/bovine/Mississip .......... .......... .......... .......... .......... .......... .......V.. .........A .......... ..........
QKU36146HEF(D/bovine/Mississip .......... .......... .......... .......... .......... ...D...... .......... .......A.. ...M...... ..........
AIO11648HEF(D/bovine/Kansas/1- .......... .......... .......... .......... .......... .......... .......... .......... ........S. ..........
AIO11624HEF(D/bovine/Kansas/14 .......... .......... .......... .......... .......... .......... .......... .......N.. .......... ..........
AIO11632HEF(D/bovine/Kansas/13 .......... .......... .......... .......... .......... .......... .......V.. .........A .......... ..........
AIO11616HEF(D/bovine/Kansas/11 .......... .......... .......... .......... .......... .......... .......V.. .........A .......... ..........
AIO11640HEF(D/bovine/Texas/3-1 .......... .......... .......... .......... .......... .......... .......V.. .........A .......... ..........
QLJ57712HEF(D/bovine/Texas/72/ .......... .......... .......... .......... .......... .......... .......... .......... .......... ..........
AIO11656HEF(D/bovine/Nebraska/ .......... .......... .......... .......... .......... .......... .......V.. .........A .......... ..........
ALE66284HEF(D/bovine/Italy/1/2 .......... .......... .......... .......... .........I .......... .......... .......N.. .......... ..........
AON76703HEF(D/swine/Italy/2545 .......... .......... .......... .......... .......... .......... .......... .......N.. .......... ..........
ALE66288HEF(D/bovine/Italy/464 .......... .......... .......... .......... .......... .......... .......... .......N.. .......... ..........
ALE66296HEF(D/swine/Italy/1997 .......... ......K... .......... .......... .......... .......... .......... .......N.. .......... ..........
AON76687HEF(D/swine/Italy/2683 .......... .......... .......... .......... .......... .......... .......... .......N.. .......... ..........
AON76711HEF(D/swine/Italy/3540 .......... .......... .......... .......... .......... .......... .......... .......N.. .......... ..........
AON76695HEF(D/swine/Italy/1732 .......... .......... .......... .......... .......... .......... .......... .......N.. .......... ..........
QDP14212HEF(D/bovine/Italy/19R .......... .......... .......... .......... .......... .......... .V.....V.. .........A .......... ..........
QDP14340HEF(D/bovine/Italy/108 .......... .......... .......... .......... .......... .......... .......... .......N.. .......... ..........
QDZ58879HEF(D/bovine/Italy/281 .......... .......... .......... ..F....... .......... .......... .......V.. .........A .......... ..........
QDZ58887HEF( D/bovine/Italy/28 .......... .......... .......... ..F....... .......... .......... .......V.. .........A .......... ..........
AUO38022HEF(D/bovine/France/59 .......... .......... .......... .......... .......... .......... .......... .......N.. .......... ..........
CEE50066HEF(D/bovine/France/29 .......... .......... .......... .......... .......... .......... .......... .........A .......... ..........
AIE52116HEF(D/bovine/Shandong/ .......... .......... .......... .......... .......... .......... .......... .......... .......... ..........
AIE52100HEF(D/bovine/Shandong/ .......... .......... .......... .......... .......... .......... .......... .......... .......... ..........
AWM99804HEF(D/bovine/Guangdong .......... .......... .......... .......... .......... .......... .......... .......... .......... ..........
AWM99802HEF(D/swine/Guangdong/ .......... .......... .......... .......... .......... .......... .......... .......... .......... ..........
AWM99806HEF(D/bovine/Guangdong .......... .......... .......... .......... .......... .......... .......... .......... .......... ..........
BBM60897HEF[D/bovine/Yamagata/ .......... .......... .......... .......... ...T...... .......... ....G..... .......N.. .......... V.........
ALR82078HEF(D/bovine/Mexico/S5 .......... .......... .......... .......... .......... .......... .......V.. .........A .......... ..........
ALR82076HEF(D/bovine/Mexico/S7 .......... .......... .......... .......... .......... .......... .......V.. .........A .......... ..........
BAY00632HEF(D/bovine/Miyazaki/ ..F....... M......K.. .......... .......... .......... .......... .......... .......... ........S. ..........
BAV17997HEF (D/bovine/Ibaraki/ .......... M......K.. .......... .......... .......... .......... .......... .......... ........S. ..........
QJP22178HEF(D/bovine/Quebec/3M .......... .......... .......... .......... .......... .......... .......V.. .......N.. .......... ..........
QJP22170HEF(D/bovine/Quebec/3E .......... .......... .......... .......... ....E..... .......... .......V.. .........A .......... ..........
C-Johannesburg-1-1966_HEF      ..FS.LLVLG L.EAEKIKIC LQKQVNSSF. LHNGF.GNLY ATEE.RMFEL VKPKAGASVL NQSTWIGF.D SRTDKSNSAF PRS.DVSAKT .DKFRSL.GG
QDG00513.1:1-651 HEF precursor ~~~~~~~~~. .......... .......... .......... .......... .......... .......... .......N.. .......... ..........
QDZ58983.. HEF precursor [Infl .......... .......... .......... .......... .......... .......... .......... .......N.. .......... ..........
AWM99805.. HEF [Influenza D vi .......... .......... .......... .......... .......... .......... .......... .......... .......... ..........
QDG00502.1:1-655 HEF precursor ~~~~~~~~~. .......... .......... .......... .......... .......... .......... .......N.. .......... ..........
AWM99801.. HEF [Influenza D vi .......... .......... .......... .......... .......... .......... .......... .......... .......... ..........
AWM99803.. HEF [Influenza D vi .......... .......... .......... .......... .......... .......... .......... .......... .......... ..........
QDZ58967.. HEF precursor [Infl .......... .......... .......... .......... .......... .......... .......V.. .........A .......... ..........
QKU36193.. HEF [Influenza D vi .......... .......... .......... .......... .......... .......... .......V.. .........A .......... ..........
QDZ58927.. HEF precursor [Infl .......... .......... .......... ..F....... .......... .......... .......V.. .........A .......... ..........
QKU36067.. HEF [Influenza D vi .......... .......... .......... .......... .......... .......... .......V.. .........A .......... ..........
AYV96999.. HEF [Influenza D vi .......... .......... .......... .......... .......... .......... .......V.. .........A .......... ..........
QDZ58943.. HEF precursor [Infl .......... .......... .......... ..F....... .......... .......... .......V.. .........A .......... ..........
QKU36035.. HEF [Influenza D vi .......... .......... .......... .......... .......... .......... .......V.. .........A .......... ..........
QKU36059.. HEF [Influenza D vi .......... .......... .......... .......... .......... .......... .......V.. .........A .......... ..........
QKU35988.. HEF [Influenza D vi .......... .......... .......... .......... .......... .......... .......V.. .........A .......... ..........
QJP22155.. HEF precursor [Infl .......... .......... .......... .......... ....E..... .......... .......V.. .........A .......... ..........
QJP22163.1:1-653 HEF precursor .......... .......... .......... .......... ....E..... .......... .......V.. .........A .......... ..........
QDG00500.. HEF precursor [Infl .......... .......... .......... .......... .......... .......... .......... .......N.. .......... ..........
QDP14324.. HEF [Influenza D vi .......... .......K.. .......... .......... .......... .......... .......... .......N.. ........XX XXXXXXXXXX
```

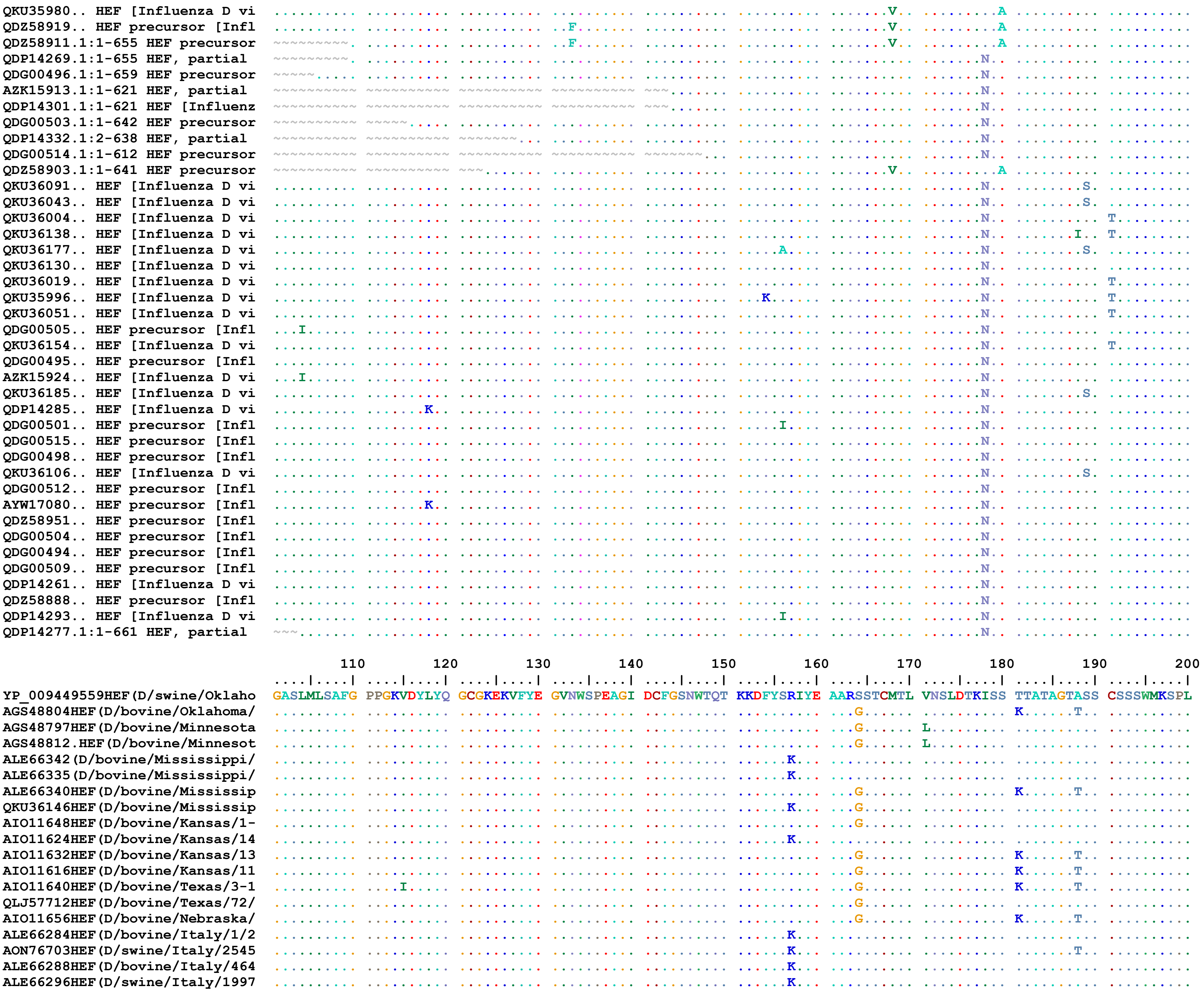

QKU35980.. HEF [Influenza D vi
QDZ58919.. HEF precursor [Infl
QDZ58911.1:1-655 HEF precursor
QDP14269.1:1-655 HEF, partial
QDG00496.1:1-659 HEF precursor
AZK15913.1:1-621 HEF, partial
QDP14301.1:1-621 HEF [Influenz
QDG00503.1:1-642 HEF precursor
QDP14332.1:2-638 HEF, partial
QDG00514.1:1-612 HEF precursor
QDZ58903.1:1-641 HEF precursor
QKU36091.. HEF [Influenza D vi
QKU36043.. HEF [Influenza D vi
QKU36004.. HEF [Influenza D vi
QKU36138.. HEF [Influenza D vi
QKU36177.. HEF [Influenza D vi
QKU36130.. HEF [Influenza D vi
QKU36019.. HEF [Influenza D vi
QKU35996.. HEF [Influenza D vi
QKU36051.. HEF [Influenza D vi
QDG00505.. HEF precursor [Infl
QKU36154.. HEF [Influenza D vi
QDG00495.. HEF precursor [Infl
AZK15924.. HEF [Influenza D vi
QKU36185.. HEF [Influenza D vi
QDP14285.. HEF [Influenza D vi
QDG00501.. HEF precursor [Infl
QDG00515.. HEF precursor [Infl
QDG00498.. HEF precursor [Infl
QKU36106.. HEF [Influenza D vi
QDG00512.. HEF precursor [Infl
AYW17080.. HEF precursor [Infl
QDZ58951.. HEF precursor [Infl
QDG00504.. HEF precursor [Infl
QDG00494.. HEF precursor [Infl
QDG00509.. HEF precursor [Infl
QDP14261.. HEF [Influenza D vi
QDZ58888.. HEF precursor [Infl
QDP14293.. HEF [Influenza D vi
QDP14277.1:1-661 HEF, partial
110 120 130 140 150 160 170 180 190 200
YP_009449559HEF(D/swine/Oklaho GASLMLSAFG PPGKVDYLYQ GCGKEKVFYE GVNWSPEAGI DCFGSNWTQT KKDFYSRIYE AARSSTCMTL VNSLDTKISS TTATAGTASS CSSSWMKSPL
AGS48804HEF(D/bovine/Oklahoma/
AGS48797HEF(D/bovine/Minnesota
AGS48812.HEF(D/bovine/Minnesot
ALE66342(D/bovine/Mississippi/
ALE66335(D/bovine/Mississippi/
ALE66340HEF(D/bovine/Mississip
QKU36146HEF(D/bovine/Mississip
AIO11648HEF(D/bovine/Kansas/1-
AIO11624HEF(D/bovine/Kansas/14
AIO11632HEF(D/bovine/Kansas/13
AIO11616HEF(D/bovine/Kansas/11
AIO11640HEF(D/bovine/Texas/3-1
QLJ57712HEF(D/bovine/Texas/72/
AIO11656HEF(D/bovine/Nebraska/
ALE66284HEF(D/bovine/Italy/1/2
AON76703HEF(D/swine/Italy/2545
ALE66288HEF(D/bovine/Italy/464
ALE66296HEF(D/swine/Italy/1997

```
AON76687HEF(D/swine/Italy/2683  .......... .......... .......... .......... .......... ......K... .......X.. .......... .......T.. ..........
AON76711HEF(D/swine/Italy/3540  .......... .......... .......... .......... .......... ......K... .......... .......... .......... ..........
AON76695HEF(D/swine/Italy/1732  .......... .......... .......... .......... ...L...... ......K... .......... .......... .......... ..........
QDP14212HEF(D/bovine/Italy/19R  .......... .......... .......... .......... .......... .......... ...G...... .......... K......T.. ..........
QDP14340HEF(D/bovine/Italy/108  .......... .......... .......... .......... .......... ......K... .......... .......... .......... ..........
QDZ58879HEF(D/bovine/Italy/281  .......... .......... .......... .......... .......... .......... ...G...... .......... K......T.. ..........
QDZ58887HEF( D/bovine/Italy/28  .......... .......... .......... .......... .......... .......... ...G...... .......... K......T.. ..........
AUO38022HEF(D/bovine/France/59  .......... .......... .......... .......... .......... ......K... .......... .......... ........F. ..........
CEE50066HEF(D/bovine/France/29  .......... ..D....... .......... .......... .......... .......... ...G...... .......... .......T.. ..........
AIE52116HEF(D/bovine/Shandong/  .......... .......... .......... .......... .......... ......M... ..IG...... .....I.... .......... ..........
AIE52100HEF(D/bovine/Shandong/  .......... .......... .......... .......... .......... ......M... ..KG...... .....I.... .......... ..........
AWM99804HEF(D/bovine/Guangdong  .......... .......... .......... .......... .......... ......M... ..KG...... .....I.... .......... ..........
AWM99802HEF(D/swine/Guangdong/  .......... .......... .......... .......... .......... ......M... ..KG...... .....I.... .......... ..........
AWM99806HEF(D/bovine/Guangdong  .......... .......... .......... .......... .......... ......M... ..KG...... .....I.... .......... ..........
BBM60897HEF[D/bovine/Yamagata/  .......... .......... .......... ........K. .......... .......... ...G...... .......... .......T.T ..........
ALR82078HEF(D/bovine/Mexico/S5  .......... .......... .......... ........E. .......... .......... ...G...... .......... K......T.. ..........
ALR82076HEF(D/bovine/Mexico/S7  .......... .......... .......... .......... .......... .......... ...G...... .....I.... K......T.. ..........
BAY00632HEF(D/bovine/Miyazaki/  .......... .......... .......... .......... .......... .......... ...G...... .......... .......T.. ..........
BAV17997HEF (D/bovine/Ibaraki/  .......... .......... .......... .......... .......... .......... ...G...... .......... .......T.. ..........
QJP22178HEF(D/bovine/Quebec/3M  .......... .......... .......... .......... .......... .......... ...G...... .......... .......T.. ..........
QJP22170HEF(D/bovine/Quebec/3E  .......... .......... .......... .......... .......... .......... ...G...... .......... K......T.. ..........
C-Johannesburg-1-1966_HEF       SLM.SMFGPP GKVDYL.QGC .KH.VFYEGV NWSPHAAINC YRKNWTDIKL NFQKNIYELA SQSHCMSLVN ALDKTIPLQV .AG...NCNN SFLKNPALYT
QDG00513.1:1-651 HEF precursor  .......... .......... .......... .......... .......... ......K... .......... .......... .......... ..........
QDZ58983.. HEF precursor [Infl  .......... .......... .......... .......... .......... ......K... .......... .......... .......... ..........
AWM99805.. HEF [Influenza D vi  .......... .......... .......... .......... .......... ......M... ..KG...... .....I.... .......... ..........
QDG00502.1:1-655 HEF precursor  .......... .......... .......... .......... .......... ......K... .......... .......... .......... ..........
AWM99801.. HEF [Influenza D vi  .......... .......... .......... .......... .......... ......M... ..KG...... .....I.... .......... ..........
AWM99803.. HEF [Influenza D vi  .......... .......... .......... .......... .......... ......M... ..KG...... .....I.... .......... ..........
QDZ58967.. HEF precursor [Infl  .......... .......... .......... .......... .......... .......... ...G...... .......... K......T.. ..........
QKU36193.. HEF [Influenza D vi  .......... .......... .......... .......... .......... .......... ...G...... .......... K......T.. ..........
QDZ58927.. HEF precursor [Infl  .......... .......... .......... .......... .......... .......... ...G...... .......... K......T.. ..........
QKU36067.. HEF [Influenza D vi  .......... .......... .......... .......... .......... .......... ...G...... .......... K......T.. ..........
AYV96999.. HEF [Influenza D vi  .......... .......... .......... .......... .......... .......... ...G...... .....I.... K......T.. ..........
QDZ58943.. HEF precursor [Infl  .......... .......... .......... .......... .......... .......... ...G...... .......... K......T.. ..........
QKU36035.. HEF [Influenza D vi  .......... .......... .......... .......... .......... .......... ...G...... .......... K......T.. ..........
QKU36059.. HEF [Influenza D vi  .......... .......... .......... .......... .......... .......... ...G...... .......... K......T.. ..........
QKU35988.. HEF [Influenza D vi  .......... .......... .......... .......... .......... .......... ...G...... .......... K......T.. ..........
QJP22155.. HEF precursor [Infl  .......... .......... .......... .......... .......... .......... ...G...... .......... K......T.. ..........
QJP22163.1:1-653 HEF precursor  .......... .......... .......... .......... .......... .......... ...G...... .......... K......T.. ..........
QDG00500.. HEF precursor [Infl  .......... .......... .......... .......... .......... ......KM.. .......... .......... .......... ..........
QDP14324.. HEF [Influenza D vi  XXXXXXXXXX XXXXXXXXXX XXXXXXXXXX X......... ......I... ......K... .......... .......... .......... ..........
QKU35980.. HEF [Influenza D vi  .......... .......... .......... .......... .......... .......... ...G...... .......... K......T.. ..........
QDZ58919.. HEF precursor [Infl  .......... .......... .......... .......... .......... .......... ...G...... .......... K......T.. ..........
QDZ58911.1:1-655 HEF precursor  .......... .......... .......... .......... .......... .......... ...G...... .......... K......T.. ..........
QDP14269.1:1-655 HEF, partial   .......... .......... .......... .......... .......... ......K... .......... .......... .......... ..........
QDG00496.1:1-659 HEF precursor  .......... .......... .......... .......... .......... ......K... .......... .......... .......... ..........
AZK15913.1:1-621 HEF, partial   .......... .......... .......... .......... .......... ......K... .......... .......... .......... ..........
QDP14301.1:1-621 HEF [Influenz  .......... .......... .......... .......... .......... ......K... .......... .......... .......... ..........
QDG00503.1:1-642 HEF precursor  .......... .......... .......... .......... .......... ......K... .......... .......... .......... ..........
QDP14332.1:2-638 HEF, partial   .......... .......... .......... .......... .......... ......K... .......... .......... .......... ..........
QDG00514.1:1-612 HEF precursor  .......... .......... .......... .......... .......... ......K... .......... .......... .......... ..........
QDZ58903.1:1-641 HEF precursor  .......... .......... .......... .......... .....XXX.. .......... ...G...... .......... K......T.. ..........
QKU36091.. HEF [Influenza D vi  .......... .......... .......... .......... .......... .......... .......... .......... .......... ..........
QKU36043.. HEF [Influenza D vi  .......... .......... .......... .......... .......... .......... .......... .......... .......... ..........
QKU36004.. HEF [Influenza D vi  .......... .......... .......... .......... .......... ......K... .......... .......... .......... ..........
QKU36138.. HEF [Influenza D vi  .......... .......... .......... .......... .......... ......K... .......... .......... .......... ..........
QKU36177.. HEF [Influenza D vi  .......... .......... .......... .......... .......... .......... .......... .......... .......... ..........
QKU36130.. HEF [Influenza D vi  .......... .......... .......... .......... .......... .......... .......... .......... .......T.. ..........
QKU36019.. HEF [Influenza D vi  .......... .......... .......... .......... .......... ......K... .......... .......... .......... ..........
QKU35996.. HEF [Influenza D vi  .......... .......... .......... .......... .......... ......K... .......... .......... .......... ..........
QKU36051.. HEF [Influenza D vi  .......... .......... .......... .......... .......... ......K... .......... .......... .......... ..........
QDG00505.. HEF precursor [Infl  .......... .......... .......... .......... .......... ......K... .......... .......... .......... ..........
```

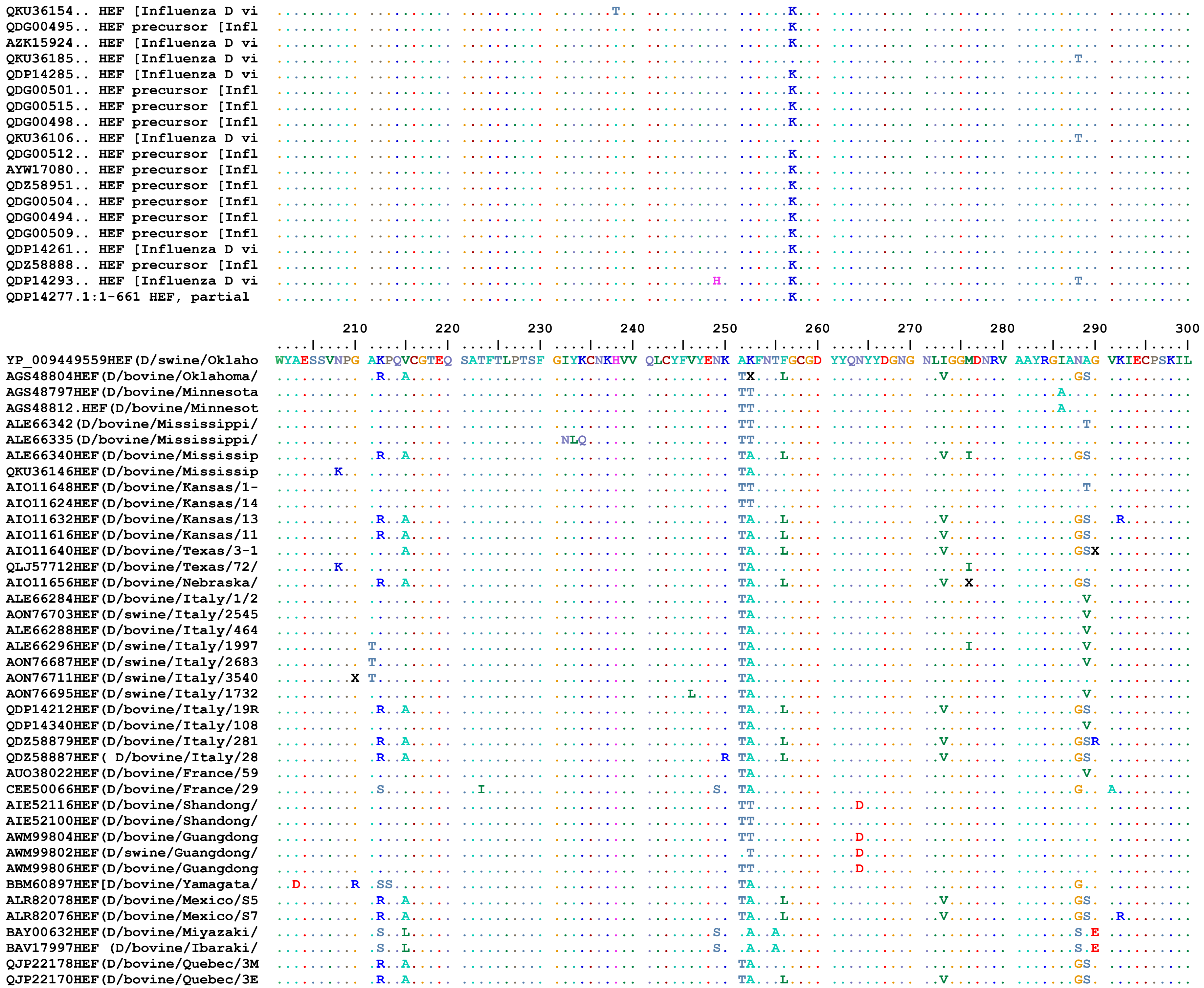

QKU36154.. HEF [Influenza D vi
QDG00495.. HEF precursor [Infl
AZK15924.. HEF [Influenza D vi
QKU36185.. HEF [Influenza D vi
QDP14285.. HEF [Influenza D vi
QDG00501.. HEF precursor [Infl
QDG00515.. HEF precursor [Infl
QDG00498.. HEF precursor [Infl
QKU36106.. HEF [Influenza D vi
QDG00512.. HEF precursor [Infl
AYW17080.. HEF precursor [Infl
QDZ58951.. HEF precursor [Infl
QDG00504.. HEF precursor [Infl
QDG00494.. HEF precursor [Infl
QDG00509.. HEF precursor [Infl
QDP14261.. HEF [Influenza D vi
QDZ58888.. HEF precursor [Infl
QDP14293.. HEF [Influenza D vi
QDP14277.1:1-661 HEF, partial
210 220 230 240 250 260 270 280 290 300
YP_009449559HEF(D/swine/Oklaho
WYAESSVNPG AKPQVCGTEQ SATFTLPTSF GIYKCNKHVV QLCYFVYENK AKFNTFGCGD YYQNYYDGNG NLIGGMDNRV AAYRGIANAG VKIECPSKIL
AGS48804HEF(D/bovine/Oklahoma/
AGS48797HEF(D/bovine/Minnesota
AGS48812.HEF(D/bovine/Minnesot
ALE66342(D/bovine/Mississippi/
ALE66335(D/bovine/Mississippi/
ALE66340HEF(D/bovine/Mississip
QKU36146HEF(D/bovine/Mississip
AIO11648HEF(D/bovine/Kansas/1-
AIO11624HEF(D/bovine/Kansas/14
AIO11632HEF(D/bovine/Kansas/13
AIO11616HEF(D/bovine/Kansas/11
AIO11640HEF(D/bovine/Texas/3-1
QLJ57712HEF(D/bovine/Texas/72/
AIO11656HEF(D/bovine/Nebraska/
ALE66284HEF(D/bovine/Italy/1/2
AON76703HEF(D/swine/Italy/2545
ALE66288HEF(D/bovine/Italy/464
ALE66296HEF(D/swine/Italy/1997
AON76687HEF(D/swine/Italy/2683
AON76711HEF(D/swine/Italy/3540
AON76695HEF(D/swine/Italy/1732
QDP14212HEF(D/bovine/Italy/19R
QDP14340HEF(D/bovine/Italy/108
QDZ58879HEF(D/bovine/Italy/281
QDZ58887HEF(D/bovine/Italy/28
AUO38022HEF(D/bovine/France/59
CEE50066HEF(D/bovine/France/29
AIE52116HEF(D/bovine/Shandong/
AIE52100HEF(D/bovine/Shandong/
AWM99804HEF(D/bovine/Guangdong
AWM99802HEF(D/swine/Guangdong/
AWM99806HEF(D/bovine/Guangdong
BBM60897HEF[D/bovine/Yamagata/
ALR82078HEF(D/bovine/Mexico/S5
ALR82076HEF(D/bovine/Mexico/S7
BAY00632HEF(D/bovine/Miyazaki/
BAV17997HEF (D/bovine/Ibaraki/
QJP22178HEF(D/bovine/Quebec/3M
QJP22170HEF(D/bovine/Quebec/3E

```
C-Johannesburg-1-1966_HEF        QEVKP.E.KC G.ENLAFFTL PTQ.GTYECK LHLVASCYFI YDSKE..NKR GCD.Y.QVIY DSSGKVV.GL DNRVSPYTGN SGDTPTMQCD MLQLK.GRYS
QDG00513.1:1-651 HEF precursor   .......... .......... .......... .......... .......... TA........ .......... .......... ........V. ..........
QDZ58983.. HEF precursor [Infl   .......... .......... .......... .......... .......... TA........ .......... .....I.... ........V. ..........
AWM99805.. HEF [Influenza D vi   .......... .......... .......... .......... .......... TT........ ...D...... .......... .......... ..........
QDG00502.1:1-655 HEF precursor   .......... .......... .......... .......... .......... TA........ .......... .......... ........V. ..........
AWM99801.. HEF [Influenza D vi   .......... .......... .......... .......... .......... .T........ ...D...... .......... .......... ..........
AWM99803.. HEF [Influenza D vi   .......... .......... .......... .......... .......... TT........ ...D...... .......... .......... ..........
QDZ58967.. HEF precursor [Infl   .......... .R..A..... .......... .......... .......... TA...L.... .......... ..V....... .......GS. ..........
QKU36193.. HEF [Influenza D vi   .......... .R..A..... .......... .......... .......... TA...L.... .......... ..V....... .......GS. ..........
QDZ58927.. HEF precursor [Infl   .......... .R..A..... .......... .......... .......... TA...L.... .......... ..V..I.... .......GS. ..........
QKU36067.. HEF [Influenza D vi   .......... .R..A..... .......... .......... .......... TA...L.... .......... .......... .......GS. ..........
AYV96999.. HEF [Influenza D vi   .......... .R..A..... .......... .......... .......... TA...L.... .......... .......... .......GSR ..........
QDZ58943.. HEF precursor [Infl   .......... .R..A..... .......... .......... .......... TA...L.... .......... ..V....... .......GS. ..........
QKU36035.. HEF [Influenza D vi   .......... .R..A..... .......... .......... .......... TA...L.... .......... ..V....... .......GS. ..........
QKU36059.. HEF [Influenza D vi   .......... .R..A..... .......... .......... .......... TA...L.... .......... ..V....... .......GS. ..........
QKU35988.. HEF [Influenza D vi   .......... .R..A..... .......... .......... .......... TA...L.... .......... ..V..X.... .......GS. ..........
QJP22155.. HEF precursor [Infl   .......... .R..A..... .......... .......... .......... TA...T.... .......... .PV..T.... .......GS. ..........
QJP22163.1:1-653 HEF precursor   .......... .R..A..... .......... .......... .......... KA...L.... .......... ..V....... .......GS. ..........
QDG00500.. HEF precursor [Infl   .......... .......... .......... .......... .......... TA........ .......... .......... .....T..V. ..........
QDP14324.. HEF [Influenza D vi   .......... .......... .......... .......... .......... TA........ .......... .....I.... ........V. ..........
QKU35980.. HEF [Influenza D vi   .......... .R..A..... .......... .......... .......... TA...L.... .......... ..V..I.... .......GS. ..........
QDZ58919.. HEF precursor [Infl   .......... .R..A..... .......... .......... .......... TA...L.... .......... ..V....... .......GS. ..........
QDZ58911.1:1-655 HEF precursor   .......... .R..A..... .......... .......... .......... TA...L.... .......... ..V....... .......GS. ..........
QDP14269.1:1-655 HEF, partial    .......... T......... .......... .......... .......... TA........ .......... .....I.... .....T..V. ..........
QDG00496.1:1-659 HEF precursor   .......... .......... .......... .......... .......... TA........ .......... .......... ........V. ..........
AZK15913.1:1-621 HEF, partial    .......... T......... .......... .......... .......... TA........ ........K. .......... .......... ..........
QDP14301.1:1-621 HEF [Influenz   .......... .......... .......... .......... .......... TA........ .......... .......... ........V. ..........
QDG00503.1:1-642 HEF precursor   .......... T......... .......... .......... .......... TA........ .......... .......... .......... ..........
QDP14332.1:2-638 HEF, partial    G......... ....I..... .........X XXXXXXXX.. .......... TA........ .......... .......... ........V. ..........
QDG00514.1:1-612 HEF precursor   .......... .......... .......... .......... .......... TA........ .......... .......... ........V. ..........
QDZ58903.1:1-641 HEF precursor   .......... ....A..... .......... .........A .......... TA...L.... .......... ..V....... .......GSR ..........
QKU36091.. HEF [Influenza D vi   .......... .......... .......... .......... .......... TT........ .......... .......... .......... ..........
QKU36043.. HEF [Influenza D vi   .......... .......... .......... .......... .......... TT........ .......... .......... .......... ..........
QKU36004.. HEF [Influenza D vi   .......... .......... .......... .......... .......... TT........ .......... .......... .......... ..........
QKU36138.. HEF [Influenza D vi   .......... .......... .......... .......... .......... TT........ .......... .......... .......... ..........
QKU36177.. HEF [Influenza D vi   .......... .......... .......... .......... .......... TT........ .......... .......... .......... ..........
QKU36130.. HEF [Influenza D vi   .......... .......... .......... .......... .......... TT........ .......... .......... .......... ..........
QKU36019.. HEF [Influenza D vi   .......... .......... .......... .......... .......... TT........ .......... .....I.... .......... ..........
QKU35996.. HEF [Influenza D vi   .......... .......... .......... .......... .......... TT........ .......... .......... .......... ..........
QKU36051.. HEF [Influenza D vi   .......... .......... .......... .......... .......... TT........ .......... .....I.... .......... ..........
QDG00505.. HEF precursor [Infl   .......... .......... .......... .......... .......... TA........ .......... .......... ........V. ..........
QKU36154.. HEF [Influenza D vi   .......... .......... .......... .......... .......... TT........ .......... .......... .......... ..........
QDG00495.. HEF precursor [Infl   .......... .......... .......... .......... .......... TA........ .......... .......... ........V. ..........
AZK15924.. HEF [Influenza D vi   .......... .......... .......... .......... .......... TA........ .......... .......... ........V. ..........
QKU36185.. HEF [Influenza D vi   .......... .......... .......... .......... .......... TT........ .......... .......... .......... ..........
QDP14285.. HEF [Influenza D vi   .......... .......... .......... .......... .......... TA........ .......... .......... ........V. ..........
QDG00501.. HEF precursor [Infl   .......... .......... .......... .......... .......... TA........ .......... .......... ........V. ..........
QDG00515.. HEF precursor [Infl   .......... T......... .......... .......... .......... TA........ .......... .....I.... ........V. ..........
QDG00498.. HEF precursor [Infl   .......... T......... .......... .......... .......... TA........ .......... .......... .......... ..........
QKU36106.. HEF [Influenza D vi   .......... .......... .......... .......... .......... TT........ .......... .....I.... .......... ..........
QDG00512.. HEF precursor [Infl   .......... T......... .......... .......... .......... TA........ .......... .......... .......... ..........
AYW17080.. HEF precursor [Infl   .......... .......... .......... .......... .......... TA........ .......... .......... ........V. ..........
QDZ58951.. HEF precursor [Infl   .......... T......... .......... .......... .......... TA........ .......... .......... .....T..V. ..........
QDG00504.. HEF precursor [Infl   .......... X......... .......... .......... .......... TA........ .......... .......... ........X. ..........
QDG00494.. HEF precursor [Infl   .......... T......... .......... .......... .......... TA........ .......... .....X.... .......... ..........
QDG00509.. HEF precursor [Infl   .......... T......... .......... .......... .......... TA........ .......... .......... .......... ..........
QDP14261.. HEF [Influenza D vi   .......... T......... .......... .......... .......... TA........ .......... .......... .....T..V. ..........
QDZ58888.. HEF precursor [Infl   .......... T......... .......... .......... .......... TA........ .......... .......... .......... ..........
QDP14293.. HEF [Influenza D vi   .......... .......... .......... .......... .......... TA........ .......... .......... ........V. ..........
QDP14277.1:1-661 HEF, partial    .......... .......... .......... .......... .......... TA........ .......... .......... ........V. ..........

                                          310        320        330        340        350        360        370        380        390        400
```

```
                              ....|....| ....|....| ....|....| ....|....| ....|....| ....|....| ....|....| ....|....| ....|....| ....|....|
YP_009449559HEF(D/swine/Oklaho NPGTYSIKST PRFLLVPKRS YCFDTDGGYP IQVVQSEWSA SRRSDNATEE ACLQTEGCIF IKKTTPYVGE ADDNHGDIEM RQLLSGLGNN DTVCVSQSGY
AGS48804HEF(D/bovine/Oklahoma/ .......R.. .K........ .......... .......... .......... .......... .......... .......... .......... ..........
AGS48797HEF(D/bovine/Minnesota .......R.. .......... .......... .......... .......... .......... .......... .......... .......... ..........
AGS48812.HEF(D/bovine/Minnesot .......R.. .......... .......... .......... .......... .......... .......... .......... .......... ..........
ALE66342(D/bovine/Mississippi/ .......R.. .......... .......... .......... .......... .......... .......... .......... .......... ..........
ALE66335(D/bovine/Mississippi/ .......R.. .......... .......... .......... .......... .......... .......... .......... .......... ..........
ALE66340HEF(D/bovine/Mississip .......R.. .K........ .......... .......... .......... .......... .......... .......... .......... ..I.......
QKU36146HEF(D/bovine/Mississip .......R.. .......... .......... .......... .......... .......... .......... .......... .......... ..........
AIO11648HEF(D/bovine/Kansas/1- .......R.. .......... .......... .......... .......... .......... .......... .......... .......... ..........
AIO11624HEF(D/bovine/Kansas/14 .......R.. .......... .......... .......... .......... .......... .......... .......... .......... ..........
AIO11632HEF(D/bovine/Kansas/13 .....R.R.. .K........ .......... .......... .......... .......... .......... .......... .......... ..........
AIO11616HEF(D/bovine/Kansas/11 .......R.. .K........ .......... .......... .......I.. .......... .......... .......... .......... ..........
AIO11640HEF(D/bovine/Texas/3-1 .......R.. .K........ .......... .......... .......... .......... .......... .G........ .......... ..........
QLJ57712HEF(D/bovine/Texas/72/ D......R.. .......... .......... .......... .......... .......... .......... .......... .......... ..........
AIO11656HEF(D/bovine/Nebraska/ .......X.. .K........ .......... .......... .......... .......... .......... .......... .......... ..........
ALE66284HEF(D/bovine/Italy/1/2 .......R.. .......... .......... .......... .......... .......... .......... .......... .......... ..........
AON76703HEF(D/swine/Italy/2545 .......... .......... .......... .......... .......... .......... .......... .......... .......... ..........
ALE66288HEF(D/bovine/Italy/464 .......R.. .......... .......... .......... .......... .......... .......... .......... .......... ..........
ALE66296HEF(D/swine/Italy/1997 .......R.. .......... .......... .......... .......... .......... .......... .......... .......... ..........
AON76687HEF(D/swine/Italy/2683 .......... .......... .......... .......... .......... .......... .......... .......... .......... ..........
AON76711HEF(D/swine/Italy/3540 .......X.. .......... .......... .......... .......X.. .......... .......... .......... ....N..... ..........
AON76695HEF(D/swine/Italy/1732 .......... .......... .......... .........V .......... .......... .......... .......... .......... ..........
QDP14212HEF(D/bovine/Italy/19R .......R.. .K........ .......... .......... .......... .......... .......... .......... .......... ..........
QDP14340HEF(D/bovine/Italy/108 .......R.. .......... .......... .......... .......... .......... .......... .......... .......... .........H
QDZ58879HEF(D/bovine/Italy/281 .......R.. .K........ .......... .......... .......... .......... .......... .......... .......... ..........
QDZ58887HEF( D/bovine/Italy/28 .......R.. .K........ .......... .......... .......... .......... .......... .......... .......... ..........
AUO38022HEF(D/bovine/France/59 .......R.. .......... .......... .......... .......... .......... .......... .......... .......... ..........
CEE50066HEF(D/bovine/France/29 .......R.. .......... .......... .......... .......... .......... .......... .......... .......... ..........
AIE52116HEF(D/bovine/Shandong/ .......R.. .......... .......... .......... .......... .......... .......... .......... .......S.. ..........
AIE52100HEF(D/bovine/Shandong/ .......R.. .......... .......... .......... .......... .......... .......A.. .......... .......S.. ..........
AWM99804HEF(D/bovine/Guangdong .......R.. .......... .......... .......... .......... .......... .......A.. .......... .......S.. ..........
AWM99802HEF(D/swine/Guangdong/ .......R.. .......... .......... .......... .......... .......... .......A.. .......... .......S.. ..........
AWM99806HEF(D/bovine/Guangdong .......R.. .......... .......... .......... .....H.... .......... .......A.. .......... .......S.. ..........
BBM60897HEF[D/bovine/Yamagata/ R......R.. .......... .......... .......... .......... .......... .......... .......T.. ........S. ..........
ALR82078HEF(D/bovine/Mexico/S5 .......R.. .K........ .......... .......... .......... .......... .......... .......... .......... ..........
ALR82076HEF(D/bovine/Mexico/S7 .......R.. .K........ .......... .......... .......... .......... .......... .......... .......S.. ..........
BAY00632HEF(D/bovine/Miyazaki/ S......R.. .K........ .......... .......... .......... .......... .......... .......... ....N..S.. ..........
BAV17997HEF (D/bovine/Ibaraki/ S......R.. .K........ .......... .......... .......... .......... .......... .......... ....N..D.. ..........
QJP22178HEF(D/bovine/Quebec/3M .......R.. .......... .......... .......... .......... .......... .......... .......... .......... ..........
QJP22170HEF(D/bovine/Quebec/3E .......R.. .K........ .......... .......... .......... .......... .......... .......... .......... ..........
C-Johannesburg-1-1966_HEF      VRSSPRFLLM .ERSYCFDMK EKGPVTAVQS .WGKGR.SDY AVDQACLSTP G.MLIQKQKP YIGEADDHHG DQEMRELLSG LDYEARCISQ SGWVNET.PF
QDG00513.1:1-651 HEF precursor .......R.. .......... .......... .......... .......... .......... .......... .......... .......... ..........
QDZ58983.. HEF precursor [Infl .......R.. .......... .......... .......... .......... .......... .......... .......... .......... .........H
AWM99805.. HEF [Influenza D vi .......R.. .......... .......... ..I....... .......... .......... .......A.. .......... .......S.. ..........
QDG00502.1:1-655 HEF precursor .......R.. .......... .......... .......... .......... .......... .......... .......... .......... .........H
AWM99801.. HEF [Influenza D vi .......R.. .......... .......... .......... .......... .......... .......A.. .......... .......S.. ..........
AWM99803.. HEF [Influenza D vi .......R.. .......... .......... .......... .......... .......... .......A.. .......... .......S.. ..........
QDZ58967.. HEF precursor [Infl .......R.. .K........ .......... .......... .......... .......... .......... .......... .......... ..........
QKU36193.. HEF [Influenza D vi D......... .K........ .......... .......... .......... .......... .......... .......... .......... ..........
QDZ58927.. HEF precursor [Infl .......R.. .K........ .......... .......... .......... .......... .......... .......... .......... ..........
QKU36067.. HEF [Influenza D vi D......R.. .K........ .......... .......... .......... .......... .......... .......... .......... ..........
AYV96999.. HEF [Influenza D vi .......R.. .K........ .......... .......... .......... .......... .......... .......T.. .......... ..........
QDZ58943.. HEF precursor [Infl .......R.. .K........ .......... .......... .......... .......... .......... .......... .......S.. ..........
QKU36035.. HEF [Influenza D vi D......R.. .K........ .......... .......... .......... .......... .......... .......... .......... ..........
QKU36059.. HEF [Influenza D vi .......R.. .K........ .......... .......... .......... .......... .......... .......... .......... ..I.......
QKU35988.. HEF [Influenza D vi D......R.. .K........ .......... .......... .......... .......... .......... .......... .......... ..........
QJP22155.. HEF precursor [Infl .......R.. .K........ .......... .......... .......... .......... .......... .......... .......... ..........
QJP22163.1:1-653 HEF precursor .......R.. .K........ .......... .......... .......... ...R...... .......... .......... .......... ..........
QDG00500.. HEF precursor [Infl .......R.. .K........ .......... .......... .......... .......... .......... .......... ........D. .........H
QDP14324.. HEF [Influenza D vi .......R.. .......... .......... .......... .......... .......... .......... .......... .......... ..........
QKU35980.. HEF [Influenza D vi D......R.. .K........ .......... .......... .......... .......... .......... .......... .......... ..........
```

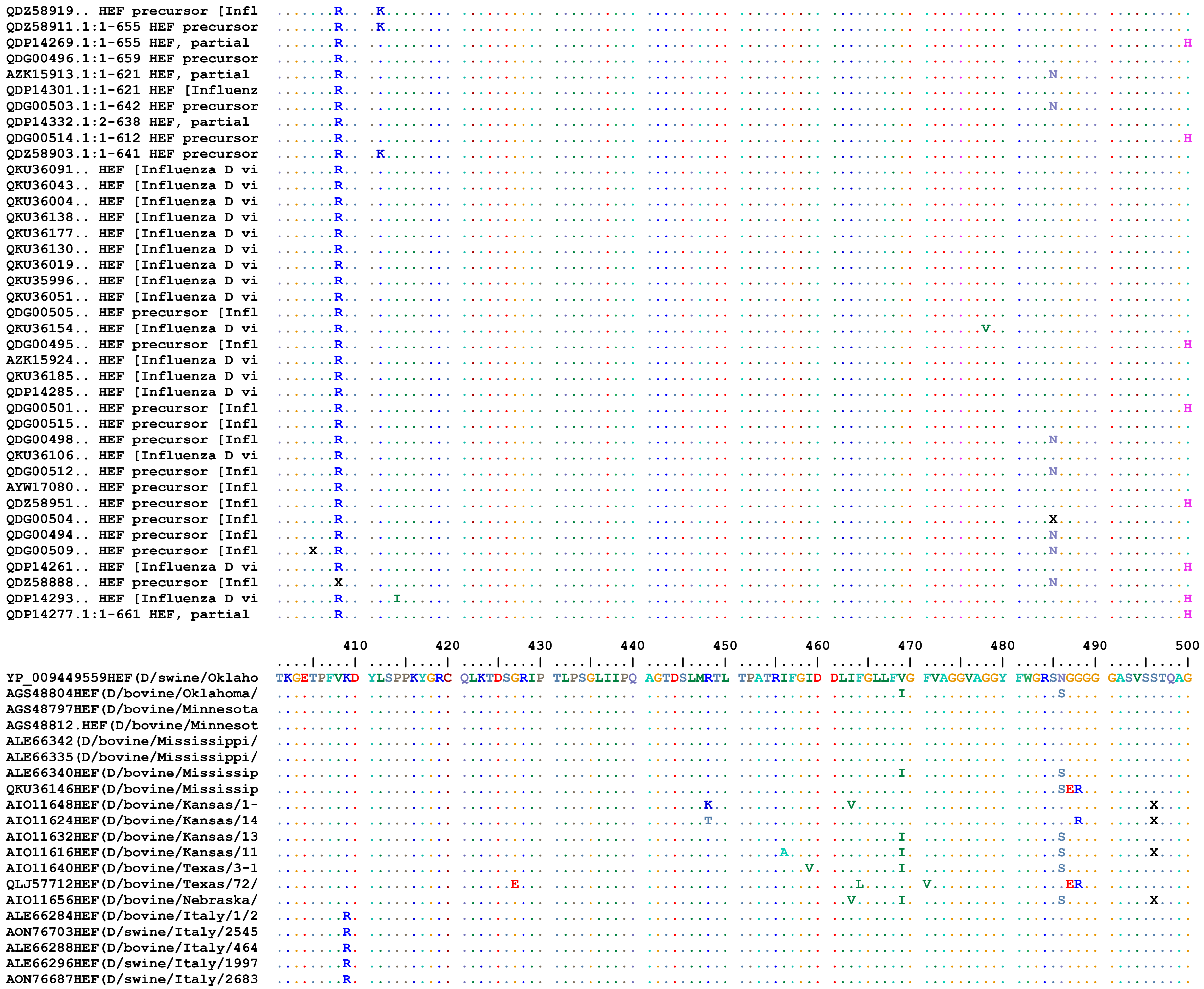

QDZ58919.. HEF precursor [Infl
QDZ58911.1:1-655 HEF precursor
QDP14269.1:1-655 HEF, partial
QDG00496.1:1-659 HEF precursor
AZK15913.1:1-621 HEF, partial
QDP14301.1:1-621 HEF [Influenz
QDG00503.1:1-642 HEF precursor
QDP14332.1:2-638 HEF, partial
QDG00514.1:1-612 HEF precursor
QDZ58903.1:1-641 HEF precursor
QKU36091.. HEF [Influenza D vi
QKU36043.. HEF [Influenza D vi
QKU36004.. HEF [Influenza D vi
QKU36138.. HEF [Influenza D vi
QKU36177.. HEF [Influenza D vi
QKU36130.. HEF [Influenza D vi
QKU36019.. HEF [Influenza D vi
QKU35996.. HEF [Influenza D vi
QKU36051.. HEF [Influenza D vi
QDG00505.. HEF precursor [Infl
QKU36154.. HEF [Influenza D vi
QDG00495.. HEF precursor [Infl
AZK15924.. HEF [Influenza D vi
QKU36185.. HEF [Influenza D vi
QDP14285.. HEF [Influenza D vi
QDG00501.. HEF precursor [Infl
QDG00515.. HEF precursor [Infl
QDG00498.. HEF precursor [Infl
QKU36106.. HEF [Influenza D vi
QDG00512.. HEF precursor [Infl
AYW17080.. HEF precursor [Infl
QDZ58951.. HEF precursor [Infl
QDG00504.. HEF precursor [Infl
QDG00494.. HEF precursor [Infl
QDG00509.. HEF precursor [Infl
QDP14261.. HEF [Influenza D vi
QDZ58888.. HEF precursor [Infl
QDP14293.. HEF [Influenza D vi
QDP14277.1:1-661 HEF, partial
410 420 430 440 450 460 470 480 490 500
YP_009449559HEF(D/swine/Oklaho TKGETPFVKD YLSPPKYGRC QLKTDSGRIP TLPSGLIIPQ AGTDSLMRTL TPATRIFGID DLIFGLLFVG FVAGGVAGGY FWGRSNGGGG GASVSSTQAG
AGS48804HEF(D/bovine/Oklahoma/
AGS48797HEF(D/bovine/Minnesota
AGS48812.HEF(D/bovine/Minnesot
ALE66342(D/bovine/Mississippi/
ALE66335(D/bovine/Mississippi/
ALE66340HEF(D/bovine/Mississip
QKU36146HEF(D/bovine/Mississip
AIO11648HEF(D/bovine/Kansas/1-
AIO11624HEF(D/bovine/Kansas/14
AIO11632HEF(D/bovine/Kansas/13
AIO11616HEF(D/bovine/Kansas/11
AIO11640HEF(D/bovine/Texas/3-1
QLJ57712HEF(D/bovine/Texas/72/
AIO11656HEF(D/bovine/Nebraska/
ALE66284HEF(D/bovine/Italy/1/2
AON76703HEF(D/swine/Italy/2545
ALE66288HEF(D/bovine/Italy/464
ALE66296HEF(D/swine/Italy/1997
AON76687HEF(D/swine/Italy/2683

```
AON76711HEF(D/swine/Italy/3540   ........R. .......... .......... .......... .......... .......... .......... .......... .......... ..........
AON76695HEF(D/swine/Italy/1732   ..R.....R. ........K. .......... .......... .......... .......... .......... .......... .......... ..........
QDP14212HEF(D/bovine/Italy/19R   .......... .......... .......... .......... .......... .......... ........I. .......... .....S.... ..........
QDP14340HEF(D/bovine/Italy/108   ......CLR. .......... .......... .......... ......I... .......... .......... .......... .......... ..........
QDZ58879HEF(D/bovine/Italy/281   .......... .......... .......... .......... .......... .......... ........I. .......... .....S.... ..........
QDZ58887HEF( D/bovine/Italy/28   .......... .......... .......... .......... .......... .......... ........I. .......... .....S.... ..........
AUO38022HEF(D/bovine/France/59   ........R. .......... .......... .......... .......... .......... .......... .......... .......... ..........
CEE50066HEF(D/bovine/France/29   .......... .......... .......... .......... .......... .......... ........I. .......... .....SE... ..........
AIE52116HEF(D/bovine/Shandong/   .......... .......... ......R... .......... .......... .......... .......... ..T....... .......... ..........
AIE52100HEF(D/bovine/Shandong/   .......... .......... .....CR... .......... .......... .......... .......... ..T....... .......... ..........
AWM99804HEF(D/bovine/Guangdong   .......... .......... .....CR... .Q........ .......... .......... .......... ..T....... .......... ..........
AWM99802HEF(D/swine/Guangdong/   .......... .......... .....CR... .....M.... .......... .......... .......... ..T....... .......... ..........
AWM99806HEF(D/bovine/Guangdong   .......... .......... .....CR... .......... .......... .......... .......... ..T....... .......... ..........
BBM60897HEF[D/bovine/Yamagata/   .......... .......... ..R....... .......... .......... .......... .....F..I. .......... .....SA... ..........
ALR82078HEF(D/bovine/Mexico/S5   .......... .......... .......... .......... .......... .......... ........I. .......... .....S.... ..........
ALR82076HEF(D/bovine/Mexico/S7   .......... .......... ......E... .......... .......... .......... ........I. .......... .....S.... ..........
BAY00632HEF(D/bovine/Miyazaki/   .......... ..I....... ......E... .......... .......... .......... ........I. .......... .....SE... ..........
BAV17997HEF  (D/bovine/Ibaraki/  .......... ..I....... ......E... .......... .......... .......... ........I. .......... .....SE... ..........
QJP22178HEF(D/bovine/Quebec/3M   .......... .......... .......... .......... .......... ..T....... ........I. .......... .....S.... ..........
QJP22170HEF(D/bovine/Quebec/3E   .......... .......... .......... .......... .......... .......... ........I. .......... .....S.... ..........
C-Johannesburg-1-1966_HEF        .EKYLLPP.F GRC.LAAKEE SIPKIPDGLL IPT..TDTTV TKPK.RIFGI DDLIIGLLFV AIVETGIGGY LLGSRKES.G GVTKESAEK. FEKIGNDIQI
QDG00513.1:1-651 HEF precursor   ........R. .......... .....N.... .......... .......... .......... .......... .......... .......... ..........
QDZ58983.. HEF precursor [Infl   ........R. .......... .......... ......V... .......... .......... .......... .......... .......... ..........
AWM99805.. HEF [Influenza D vi   .......... .......... .....CR... .......... .......... .......... .......... ..T....... .......... ..........
QDG00502.1:1-655 HEF precursor   ........R. .......... .......... .......... .......... .......... .......... L......... .......... ..........
AWM99801.. HEF [Influenza D vi   .......... .......... .....CR... .......... .......... .......... .......... ..T....... .......... ..........
AWM99803.. HEF [Influenza D vi   .......... .......... .....CR... .......... .......... .......... .......... ..T....... .......... ..........
QDZ58967.. HEF precursor [Infl   .......... .......... .......... .......... .......... .......... ........I. .......... .....S.... ..........
QKU36193.. HEF [Influenza D vi   .......... .......... .......... .......... .......... .......... ........I. .......... .....S.... ..........
QDZ58927.. HEF precursor [Infl   .......... .......... .......... .......... .......... .......... ........I. .......... .....S.... ..........
QKU36067.. HEF [Influenza D vi   .......... .......... .......... .......... .......... .......... ........I. .......... .....S.... ..........
AYV96999.. HEF [Influenza D vi   .......... .......... .......... .......... .......... .......... ........I. .......... .....S.... ..........
QDZ58943.. HEF precursor [Infl   .......... .......... .......... .......... .......... .......... ........I. .......... .....S.... ..........
QKU36035.. HEF [Influenza D vi   .......... .......... .......... .......... .......... .......... ........I. .......... .....S.... ..........
QKU36059.. HEF [Influenza D vi   .......... .......... .......... .......... .......... .......... ........I. .......... .....S.... ..........
QKU35988.. HEF [Influenza D vi   .......... .......... .......... .......... .......... .......... ........I. .......... .....S.... ..........
QJP22155.. HEF precursor [Infl   .......... .......... .......... .......... .......... .......... ........I. .......... .....S.... ..........
QJP22163.1:1-653 HEF precursor   .......... .......... .......... .......... .......... ..T....... ........I. .......... .....S.... ..........
QDG00500.. HEF precursor [Infl   ........R. .......... .......... .......... .......... .......... .......... .......... .......... ..........
QDP14324.. HEF [Influenza D vi   ........R. .......... .......... .......... .......... ..T....... .......... .......... .......... ..........
QKU35980.. HEF [Influenza D vi   .......... ..X....... .......... .......... .......... .......... ........I. .......... .....S.... ..........
QDZ58919.. HEF precursor [Infl   .......... .......... .......... .......... .......... .......... ........I. .......... .....S.... ..........
QDZ58911.1:1-655 HEF precursor   .......... .......... .......... .......... .......... .......... ........I. .......... .....S.... ..........
QDP14269.1:1-655 HEF, partial    ........R. .......... .......... .......... .......... .......... .......... .......... .......... ..........
QDG00496.1:1-659 HEF precursor   ........R. .......... .......... .......... .......... .......... .......... .......... .......... ..........
AZK15913.1:1-621 HEF, partial    ........R. .......... .......... .......... .......... .......... .......... .......... .......... ..........
QDP14301.1:1-621 HEF [Influenz   ........R. .......... .......... .......... .......... ..T....... .......... .......... .......... ..........
QDG00503.1:1-642 HEF precursor   ........R. .......... .......... .......... .......... .......... .......... .......... .......... ..........
QDP14332.1:2-638 HEF, partial    ........R. .......... .......... .......... .......... .......... .......... .......... .......... ..........
QDG00514.1:1-612 HEF precursor   ........R. .......... .......... .......... .......... .......... .......... .......... .......... ..........
QDZ58903.1:1-641 HEF precursor   .......... .......... .......... .......... .......... .......... ........I. .......... .....S.... ..........
QKU36091.. HEF [Influenza D vi   .......... .......... .......... .......... .......... .......... .......... .......... .......... ..........
QKU36043.. HEF [Influenza D vi   .......... .......... .......... .......... .......... .......... .......... .......... .......... ..........
QKU36004.. HEF [Influenza D vi   .......... .......... .......... .......... .......... .......... .......... .......... .......... ..........
QKU36138.. HEF [Influenza D vi   .......... .......... .......... .......... .......... .......... .......... .......... .......... ..........
QKU36177.. HEF [Influenza D vi   .......... .......... .......... .......... .......... .......... .......... .......... .......... ..........
QKU36130.. HEF [Influenza D vi   .......... .......... .......... .......... .......... .......... .......... .......... .......... ..........
QKU36019.. HEF [Influenza D vi   .......... .......... .......... .......... .......... .......... .......... .......... .......... ..........
QKU35996.. HEF [Influenza D vi   .......... .......... .......... .......... .......... .......... .......... .......... .......... ..........
QKU36051.. HEF [Influenza D vi   .......... .......... .......... .......... .......... .......... .......... .......... .......... ..........
QDG00505.. HEF precursor [Infl   ........R. .......... .......... .......... .......... .......... .......... .......... .......... ..........
QKU36154.. HEF [Influenza D vi   .......... .......... .......... .......... .......... .......... .......... .......... .......... ..........
```

```
QDG00495.. HEF precursor [Infl  ........R. .......... .......... .......... .......... .......... .......... .......... .......... ..........
AZK15924.. HEF [Influenza D vi  ........R. .......... .......... .......... .......... .......... .......... .......... .......... ..........
QKU36185.. HEF [Influenza D vi  .......... .......... .......... .......... .......... .......... .......... .......... .......... ..........
QDP14285.. HEF [Influenza D vi  ........R. .......... .......... .......... .......... ..T....... .......... .......... .......... ..........
QDG00501.. HEF precursor [Infl  ........R. .......... .......... .......... .......... .......... .......... .......... .......... ..........
QDG00515.. HEF precursor [Infl  ........R. .......... .......... .......... .......... .......... .......... .......... .......... ..........
QDG00498.. HEF precursor [Infl  ........R. .......... .......... .......... .......... .......... .......... .......... .......... ..........
QKU36106.. HEF [Influenza D vi  .......... .......... .......... .......... .......... .......... .......... .......... .......... ..........
QDG00512.. HEF precursor [Infl  ........R. .......... .......... .......... .......... .......... .......... .......... .......... .....X....
AYW17080.. HEF precursor [Infl  .......... .......... .......... .......... .......... ..T....... .......... .......... .......... ..........
QDZ58951.. HEF precursor [Infl  ........R. .......... .......... .......... .......... .......... .......... .......... .......... ..........
QDG00504.. HEF precursor [Infl  ........R. .......... .......... .......... .......... .......... .......... .......... .......... ..........
QDG00494.. HEF precursor [Infl  ........R. .......... .......... .......... .......... .......... .......... .......... .......... ..........
QDG00509.. HEF precursor [Infl  ........R. .......... .......... .......... .......... .......... .......... .......... .......... ..........
QDP14261.. HEF [Influenza D vi  ........R. .......... .......... .......... .......... .......... .......... .......... .......... ..........
QDZ58888.. HEF precursor [Infl  ........R. .......... .......... .......... .......... .......... .......... .......... .......... ...X......
QDP14293.. HEF [Influenza D vi  ........R. .......... .......... .......... .......... .......... .......... .......... .......... ..........
QDP14277.1:1-661 HEF, partial   ........R. .......... .......... .......... .......... .......... .......... .......... .......... ..........

                                       510        520        530        540        550        560        570        580        590        600
                                ....|....| ....|....| ....|....| ....|....| ....|....| ....|....| ....|....| ....|....| ....|....| ....|....|
YP_009449559HEF(D/swine/Oklaho  FDKIGKDIQQ LRNDTNAAIE GFNGRIAHDE QAIKNLAKEI EDARAEALVG ELGIIRSLIV ANISMNLKES LYELANQITK RGGGIAQEAG PGCWYVDSEN
AGS48804HEF(D/bovine/Oklahoma/  .......... .......... .......... .......... .......... .......... .......... .......... .......... ..........
AGS48797HEF(D/bovine/Minnesota  .......... .......... .......... .......... .......... .......... .......... .......... .......... ..........
AGS48812.HEF(D/bovine/Minnesot  .......... .......... .......... .......... .......... .......... .......... .......... .......... .........X
ALE66342(D/bovine/Mississippi/  .......... .......... .......... .......... .......... .......... ..L....... .......... .......... ..........
ALE66335(D/bovine/Mississippi/  .......... .......... .......... .......... .......... .......... ..L....... .......... .......... ..........
ALE66340HEF(D/bovine/Mississip  .......... .......... .......... .......... .......... .......... .......... .......... .......... ..........
QKU36146HEF(D/bovine/Mississip  .......... .......V.. .......... .......... .......... .......... .......... .......... .......... ..........
AIO11648HEF(D/bovine/Kansas/1-  .......... .......... .......... .......... .......... .......... .......... .......... .......... ..........
AIO11624HEF(D/bovine/Kansas/14  .......... .......... .......... .......... .......... .......... .......... .......... .......... ..........
AIO11632HEF(D/bovine/Kansas/13  .......... .......... .......... .......... .......... .......... .......... .......... .......... ..........
AIO11616HEF(D/bovine/Kansas/11  .......... .......... .......... .......... .......... .......... .......... .......... .......... ..........
AIO11640HEF(D/bovine/Texas/3-1  .......... .......... .......... .......... .......... .......... .......... .......... .......... ..........
QLJ57712HEF(D/bovine/Texas/72/  .......... .......... .......... .......... .......... .......... .......... .........R .......... ..........
AIO11656HEF(D/bovine/Nebraska/  .......... .......... .......... .......... .......... .......... .......... .......... .......... ..........
ALE66284HEF(D/bovine/Italy/1/2  .......... .......... .......... .......... .......... .......... ..L....... .......... .......... ..........
AON76703HEF(D/swine/Italy/2545  .......... F......... .......... .......... .......... .......... ..L....... .......... .......... ..........
ALE66288HEF(D/bovine/Italy/464  .......... .......... .......... .......... .......... .......... ..L....... .......... .......... ..........
ALE66296HEF(D/swine/Italy/1997  .......... .......... .......... .......... .......... .......... ..L....... .......... .......... ..........
AON76687HEF(D/swine/Italy/2683  .......... F......... .......... .......... .......... .......... ..L....... .......... .......... ..........
AON76711HEF(D/swine/Italy/3540  .......... .......... .......... .......... .......... .......... ..L....... .......... .......... ..........
AON76695HEF(D/swine/Italy/1732  .......... .......... .......... .......... .......... .......... ..L....... .......... .......... ..........
QDP14212HEF(D/bovine/Italy/19R  .......... .......... .......... .......... .......... .......... .......... .....S.... .......... ..........
QDP14340HEF(D/bovine/Italy/108  .......... .......... .......... .......... .......... .......... ..L....... .......... .......... ..........
QDZ58879HEF(D/bovine/Italy/281  .......... .......... .......... .......... .......... .......... .......... .....S.... .......... ..........
QDZ58887HEF( D/bovine/Italy/28  .......... .......... .......... .......... .......... .......... .......... .....S.... .......... ..........
AUO38022HEF(D/bovine/France/59  .......... .......... .......... .......... .......... .......... ..L....... .H........ .......... ..........
CEE50066HEF(D/bovine/France/29  .......... ..S....... .......... ..V....... .......... .......... .......... .......... .......... ..........
AIE52116HEF(D/bovine/Shandong/  .......... .......... .......... .......... .......... .......... .......... .......... .......... ..........
AIE52100HEF(D/bovine/Shandong/  .......... .......... .......... .......... .......... .......... .......... .......... .......... ..........
AWM99804HEF(D/bovine/Guangdong  .......... .......... .......... .......... .......... .......... .......... .......... .......... ..........
AWM99802HEF(D/swine/Guangdong/  .......... .......... .......... .......... .......... .......... .......... .......... .......... ..........
AWM99806HEF(D/bovine/Guangdong  .......... .......... .......... .......... .......... .......... .......... .......... .......... ..........
BBM60897HEF[D/bovine/Yamagata/  .......... .......... .......... .......... .......... .......... .......... .......... .......... ..........
ALR82078HEF(D/bovine/Mexico/S5  .......... .......... ...R...... .......... .......... .......... .......... .......... .......... ..........
ALR82076HEF(D/bovine/Mexico/S7  .......... .......... ...R...... .......... .......... .......... .......... .......... .......... ..........
BAY00632HEF(D/bovine/Miyazaki/  .......... .......... .......... ....S..... .......... .......... .......... .......... .......... ..........
BAV17997HEF (D/bovine/Ibaraki/  .......... .......... .......... ....S..... .......... .......... .......... .......... .......... ..........
QJP22178HEF(D/bovine/Quebec/3M  .......... .......... .......... .......... .......... .......... .......... .......... .......... ..........
QJP22170HEF(D/bovine/Quebec/3E  .......... .......... .......... .......... .......... .......... .......... .......... .......... ..........
C-Johannesburg-1-1966_HEF       LKSSINIAIE KL..RISHD. QAIRDLTLEI ENARSE.LLG .LGIIR..LV GNIS.GLQES LWELASEITN RAGDLAVEVS P.CW.IDNNI CDQSCQNFIF
```

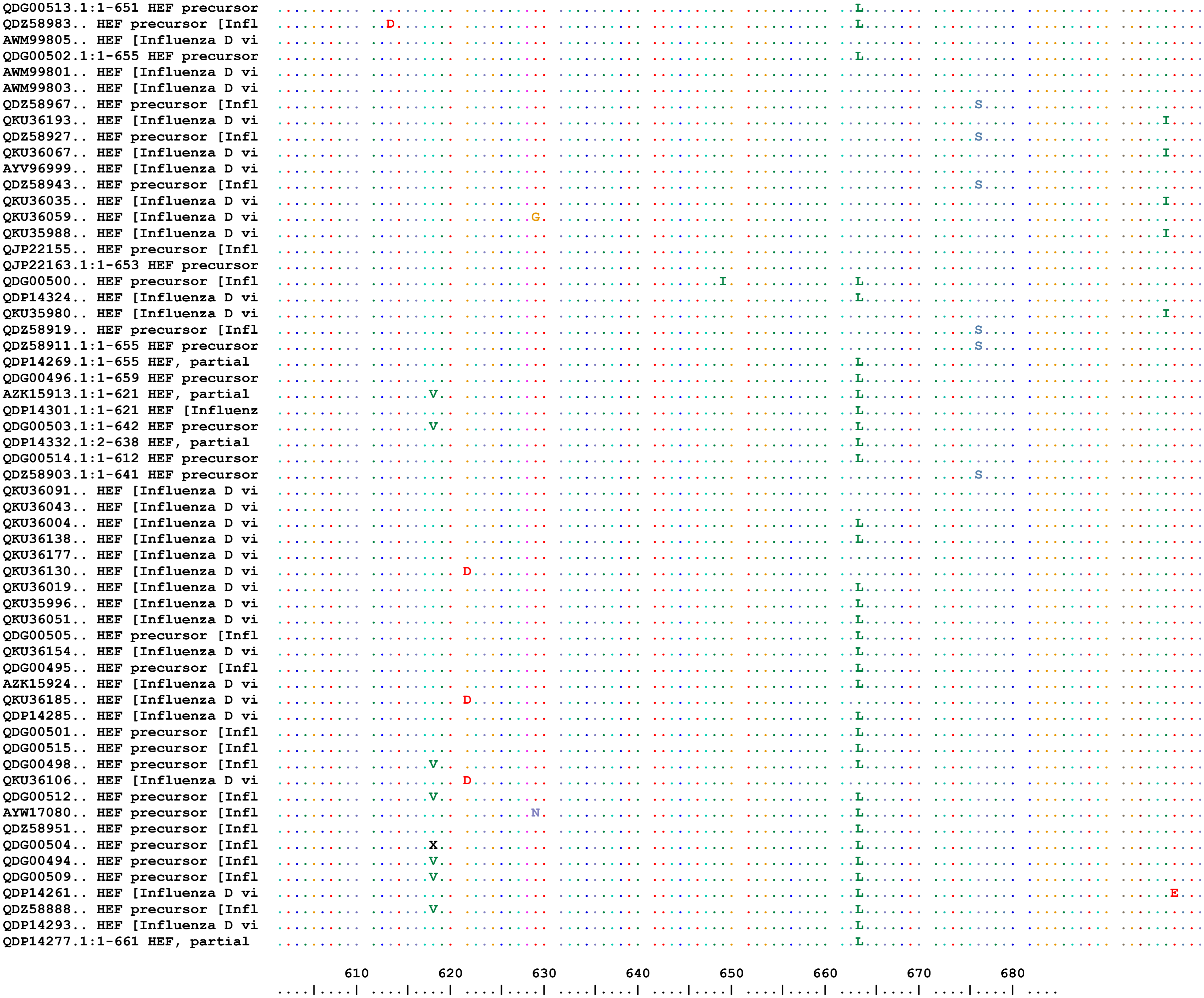
QDG00513.1:1-651 HEF precursor
QDZ58983.. HEF precursor [Infl
AWM99805.. HEF [Influenza D vi
QDG00502.1:1-655 HEF precursor
AWM99801.. HEF [Influenza D vi
AWM99803.. HEF [Influenza D vi
QDZ58967.. HEF precursor [Infl
QKU36193.. HEF [Influenza D vi
QDZ58927.. HEF precursor [Infl
QKU36067.. HEF [Influenza D vi
AYV96999.. HEF [Influenza D vi
QDZ58943.. HEF precursor [Infl
QKU36035.. HEF [Influenza D vi
QKU36059.. HEF [Influenza D vi
QKU35988.. HEF [Influenza D vi
QJP22155.. HEF precursor [Infl
QJP22163.1:1-653 HEF precursor
QDG00500.. HEF precursor [Infl
QDP14324.. HEF [Influenza D vi
QKU35980.. HEF [Influenza D vi
QDZ58919.. HEF precursor [Infl
QDZ58911.1:1-655 HEF precursor
QDP14269.1:1-655 HEF, partial
QDG00496.1:1-659 HEF precursor
AZK15913.1:1-621 HEF, partial
QDP14301.1:1-621 HEF [Influenz
QDG00503.1:1-642 HEF precursor
QDP14332.1:2-638 HEF, partial
QDG00514.1:1-612 HEF precursor
QDZ58903.1:1-641 HEF precursor
QKU36091.. HEF [Influenza D vi
QKU36043.. HEF [Influenza D vi
QKU36004.. HEF [Influenza D vi
QKU36138.. HEF [Influenza D vi
QKU36177.. HEF [Influenza D vi
QKU36130.. HEF [Influenza D vi
QKU36019.. HEF [Influenza D vi
QKU35996.. HEF [Influenza D vi
QKU36051.. HEF [Influenza D vi
QDG00505.. HEF precursor [Infl
QKU36154.. HEF [Influenza D vi
QDG00495.. HEF precursor [Infl
AZK15924.. HEF [Influenza D vi
QKU36185.. HEF [Influenza D vi
QDP14285.. HEF [Influenza D vi
QDG00501.. HEF precursor [Infl
QDG00515.. HEF precursor [Infl
QDG00498.. HEF precursor [Infl
QKU36106.. HEF [Influenza D vi
QDG00512.. HEF precursor [Infl
AYW17080.. HEF precursor [Infl
QDZ58951.. HEF precursor [Infl
QDG00504.. HEF precursor [Infl
QDG00494.. HEF precursor [Infl
QDG00509.. HEF precursor [Infl
QDP14261.. HEF [Influenza D vi
QDZ58888.. HEF precursor [Infl
QDP14293.. HEF [Influenza D vi
QDP14277.1:1-661 HEF, partial
610
620
630
640
650
660
670
680

```
YP_009449559HEF(D/swine/Oklaho  CDASCKEYIF NFNGSATVPT LRPVDTKVVI TSDPYYLGST IALCLLGLVA IAASVGVIWI CCKK------ ---------- ----
AGS48804HEF(D/bovine/Oklahoma/  .......... .......... ......N... .......... ........M. ...F...S.. ....------ ---------- ----
AGS48797HEF(D/bovine/Minnesota  .......... .......... .......... .......... .......... .......... ....------ ---------- ----
AGS48812.HEF(D/bovine/Minnesot  .......... .......... .......... .......... .......... .......... ....------ ---------- ----
ALE66342(D/bovine/Mississippi/  .......... .......... .......... .......... .......... .......... ....------ ---------- ----
ALE66335(D/bovine/Mississippi/  .......... .......... .......... .......... .......... .......... ....------ ---------- ----
ALE66340HEF(D/bovine/Mississip  .......... ...D...... ......N... .......... ........M. ...F...S.. ....------ ---------- ----
QKU36146HEF(D/bovine/Mississip  .......... .......... .......... .......... .......... ..T....... ....------ ---------- ----
AIO11648HEF(D/bovine/Kansas/1-  .......... .......... .......... .......... .......... .......... ....------ ---------- ----
AIO11624HEF(D/bovine/Kansas/14  .......... .......... .......... .......... .......... .......... ....------ ---------- ----
AIO11632HEF(D/bovine/Kansas/13  .......... .......... ......N... .......... .......... ...F...... ....------ ---------- ----
AIO11616HEF(D/bovine/Kansas/11  .......... .......... ......N... .......... M......... ...F...... ....------ ---------- ----
AIO11640HEF(D/bovine/Texas/3-1  .......... .......... ......N... .......... .......... ...F...... ....------ ---------- ----
QLJ57712HEF(D/bovine/Texas/72/  .......... .......... .......... .......... .......... .......... ....------ ---------- ----
AIO11656HEF(D/bovine/Nebraska/  .......... .......... ......N... .......... ........M. ...F...S.. ....------ ---------- ----
ALE66284HEF(D/bovine/Italy/1/2  .......... .......... .......... .......... .......... .V........ ....------ ---------- ----
AON76703HEF(D/swine/Italy/2545  .......... .......... .......... .......... .......... .V........ ....------ ---------- ----
ALE66288HEF(D/bovine/Italy/464  .......... .......... .......... .......... .......... .V........ ....------ ---------- ----
ALE66296HEF(D/swine/Italy/1997  .......... .......... .......... .......... .......... .V........ ....------ ---------- ----
AON76687HEF(D/swine/Italy/2683  .......... .......... .......... .......... .......... .V........ ....------ ---------- ----
AON76711HEF(D/swine/Italy/3540  .......... .......... .......... .......... .......... .V........ ....------ ---------- ----
AON76695HEF(D/swine/Italy/1732  .......... .......... .......... .......... .......... .V........ ....------ ---------- ----
QDP14212HEF(D/bovine/Italy/19R  .......... .......... ......N... .......... ........M. ...F...... ...R------ ---------- ----
QDP14340HEF(D/bovine/Italy/108  .......... .......... .......... .......... .......... .V........ ....------ ---------- ----
QDZ58879HEF(D/bovine/Italy/281  .......... .......... ......N... .......... ........M. ...F...... ...R------ ---------- ----
QDZ58887HEF( D/bovine/Italy/28  .......... .......... ......N... .......... ........M. ...F...... ...R------ ---------- ----
AUO38022HEF(D/bovine/France/59  .......... .......... .......... .......... .......... .V........ ....------ ---------- ----
CEE50066HEF(D/bovine/France/29  .......... .......... ......N.I. .......... .......... ...F...... ....------ ---------- ----
AIE52116HEF(D/bovine/Shandong/  .......... .......... .......... .......... .......... .......... ....------ ---------- ----
AIE52100HEF(D/bovine/Shandong/  .......... .......... .......... .......... .......... .......... ....------ ---------- ----
AWM99804HEF(D/bovine/Guangdong  .......... .......... .......... .......... .......... .......... ....------ ---------- ----
AWM99802HEF(D/swine/Guangdong/  .......... .......... .......... .......... .......... .......... ....------ ---------- ----
AWM99806HEF(D/bovine/Guangdong  .......... .......... .......... .......... .......... .......... ....------ ---------- ----
BBM60897HEF[D/bovine/Yamagata/  .......... ......P... V.T...S... .......... .......... ...F.....V ....------ ---------- ----
ALR82078HEF(D/bovine/Mexico/S5  .......... .......... ......N... .......... .......... ...F...... ....------ ---------- ----
ALR82076HEF(D/bovine/Mexico/S7  .......... .......... ......N... .......... .......... ...F...... ....------ ---------- ----
BAY00632HEF(D/bovine/Miyazaki/  ...G...... .......... ......N... .......... T......... ...F...... ....------ ---------- ----
BAV17997HEF (D/bovine/Ibaraki/  ...G...... .......... ......N... .......... T......... ...F...... ....------ ---------- ----
QJP22178HEF(D/bovine/Quebec/3M  .......... .......... ......N... .......... ........M. ...F...... ....------ ---------- ----
QJP22170HEF(D/bovine/Quebec/3E  .......... .......... ......N... .......... ........M. ...------- ---------- ---------- ----
C-Johannesburg-1-1966_HEF       KFNETAPVPT IPPLDTKIDL QSDPFYWGSS LGLAITATIS L.ALVISGI. .CRTK*LRQF *KMDNVLVNI LYSFIKNKNP LATA
QDG00513.1:1-651 HEF precursor  .......... .......... .......... .......... .......... .V..I..... ---------- ---------- ----
QDZ58983.. HEF precursor [Infl  .......... .......... .......... .......... .......... .V........ ....------ ---------- ----
AWM99805.. HEF [Influenza D vi  .......... .......... .......... .......... .......... .......... ....------ ---------- ----
QDG00502.1:1-655 HEF precursor  .......... .......... .......... .......... .......... .V........ ....------ ---------- ----
AWM99801.. HEF [Influenza D vi  .......... .......... .......... .......... .......... .......... ....------ ---------- ----
AWM99803.. HEF [Influenza D vi  .......... .......... .......... .......... .......... .......... ....------ ---------- ----
QDZ58967.. HEF precursor [Infl  .......... .......... ......N... .......... ........M. ...F...... ...R------ ---------- ----
QKU36193.. HEF [Influenza D vi  .......... .......... ......N... .......... ........M. .T.F...S.. ....------ ---------- ----
QDZ58927.. HEF precursor [Infl  .......... .......... ......N... .......... ........M. ...F...... ...R------ ---------- ----
QKU36067.. HEF [Influenza D vi  .......... .......... ......N... .......... ........M. .T.F...S.. ....------ ---------- ----
AYV96999.. HEF [Influenza D vi  .......... .......... ......N... .......... ........M. ...F...... ....------ ---------- ----
QDZ58943.. HEF precursor [Infl  .......... .......... ......N... .......... ........M. ...F...... ...R------ ---------- ----
QKU36035.. HEF [Influenza D vi  .......... .......... ......N... .......... ........M. .T.F...S.. ....------ ---------- ----
QKU36059.. HEF [Influenza D vi  .......... ...D...... ......N... .......... ........M. ...F...S.. ....------ ---------- ----
QKU35988.. HEF [Influenza D vi  .......... .......... ......N... .......... ........M. .T.F...S.. ....------ ---------- ----
QJP22155.. HEF precursor [Infl  .......... .......... ......N... .......... ........M. ...F....L. ....------ ---------- ----
QJP22163.1:1-653 HEF precursor  .......... .......... ......N... .......... ........M. ...------- ---------- ---------- ----
QDG00500.. HEF precursor [Infl  .......... .......... .......... .......... .......... .V........ ....------ ---------- ----
QDP14324.. HEF [Influenza D vi  .......... .......... .......... .......... .......... .V........ ....------ ---------- ----
QKU35980.. HEF [Influenza D vi  .......... .......... ......N... .......... ........M. .T.F...S.. ....------ ---------- ----
QDZ58919.. HEF precursor [Infl  .......... .......... ......N... .......... ........M. ...F...... ...R------ ---------- ----
```

```
QDZ58911.1:1-655 HEF precursor   .......... .......... ......N... .......... ........M. ...F...... ...R------ ---------- ----
QDP14269.1:1-655 HEF, partial    .......... .......... .......... .......... .......... .V........ ....------ ---------- ----
QDG00496.1:1-659 HEF precursor   .......... .......... .......... .......... .......... .V........ ....------ ---------- ----
AZK15913.1:1-621 HEF, partial    .......... .......... .......... .......... .......... .V........ ....------ ---------- ----
QDP14301.1:1-621 HEF [Influenz   .......... .......... .......... .......... .......... .V........ ....------ ---------- ----
QDG00503.1:1-642 HEF precursor   .......... .......... .......... .......... .......... .V.....--- ---------- ---------- ----
QDP14332.1:2-638 HEF, partial    .......... .......... .......... .......... .......... .V........ ....------ ---------- ----
QDG00514.1:1-612 HEF precursor   .......... .......... .......... .......... .......... .V.......- ---------- ---------- ----
QDZ58903.1:1-641 HEF precursor   .......... .......... ......N... .......... ........M. ...F...... ...R------ ---------- ----
QKU36091.. HEF [Influenza D vi   .......... .......... .......... .......... .......... V......... ....------ ---------- ----
QKU36043.. HEF [Influenza D vi   .......... .......... .......... .......... .......... V......V.. ....------ ---------- ----
QKU36004.. HEF [Influenza D vi   .......... .......... .......... .......... .......... .......... ....------ ---------- ----
QKU36138.. HEF [Influenza D vi   .......... .......... .......... .......... .......... .......... ....------ ---------- ----
QKU36177.. HEF [Influenza D vi   .......... .......... .......... .......... .......... V......V.. ....------ ---------- ----
QKU36130.. HEF [Influenza D vi   .......... .......... .......... .......... .......... V......V.. ....------ ---------- ----
QKU36019.. HEF [Influenza D vi   .......... .......... .......... .......... .......... .......... ....------ ---------- ----
QKU35996.. HEF [Influenza D vi   .......... .......... .......... .......... .......... .......... ....------ ---------- ----
QKU36051.. HEF [Influenza D vi   .......... .......... .......... .......... .......... T......... ....------ ---------- ----
QDG00505.. HEF precursor [Infl   .......... .......... .......... .......... .......... .V........ ....------ ---------- ----
QKU36154.. HEF [Influenza D vi   .......... .......... .......... .......... .........T .......... ....------ ---------- ----
QDG00495.. HEF precursor [Infl   .......... .......... .......... .......... .......... .V........ ....------ ---------- ----
AZK15924.. HEF [Influenza D vi   .......H.. .......... .......... .......... .......... .V........ ....------ ---------- ----
QKU36185.. HEF [Influenza D vi   .......... .......... .......... .......... .......... V......V.. ....------ ---------- ----
QDP14285.. HEF [Influenza D vi   .......... .......... .......... .......... .......... .V........ ....------ ---------- ----
QDG00501.. HEF precursor [Infl   .......... .......... .......... .......... .......... .V........ ....------ ---------- ----
QDG00515.. HEF precursor [Infl   .......... .......... .......... .......... .......... .V........ ....------ ---------- ----
QDG00498.. HEF precursor [Infl   .......... .......... .......... .......... .......... .V........ ....------ ---------- ----
QKU36106.. HEF [Influenza D vi   .......... .......... .......... .......... .......... V......V.. ....------ ---------- ----
QDG00512.. HEF precursor [Infl   .......... .......... .......... .......... .......... .V........ ....------ ---------- ----
AYW17080.. HEF precursor [Infl   .......... .......... .......... .......... .......... .V........ ....------ ---------- ----
QDZ58951.. HEF precursor [Infl   .......... .......... .......... .......... .......... .V........ ....------ ---------- ----
QDG00504.. HEF precursor [Infl   .......... .......... .......... .......... .......... .V........ ....------ ---------- ----
QDG00494.. HEF precursor [Infl   .......... .......... .......... .......... .......... .V........ ....------ ---------- ----
QDG00509.. HEF precursor [Infl   .......... .......... .......... .......... .......... .V........ ....------ ---------- ----
QDP14261.. HEF [Influenza D vi   .......... .......... .......... .......... .......... .V........ ....------ ---------- ----
QDZ58888.. HEF precursor [Infl   .......... .......... .......... .......... .......... .V........ ....------ ---------- ----
QDP14293.. HEF [Influenza D vi   .......... .......... .......... .......... .......... .V........ ....------ ---------- ----
QDP14277.1:1-661 HEF, partial    .......... .......... .......... .......... .......... .V........ ....------ ---------- ----
```
