# Supplementary material for: New Diagnostic Assays for Differential Diagnosis Between the Two Distinct Lineages of Bovine Influenza D Viruses and Human Influenza C Viruses
Source: Front Vet Sci. 2020 Dec 11;7:605704. doi: 10.3389/fvets.2020.605704 (PMC7759653; doi:10.3389/fvets.2020.605704)

A


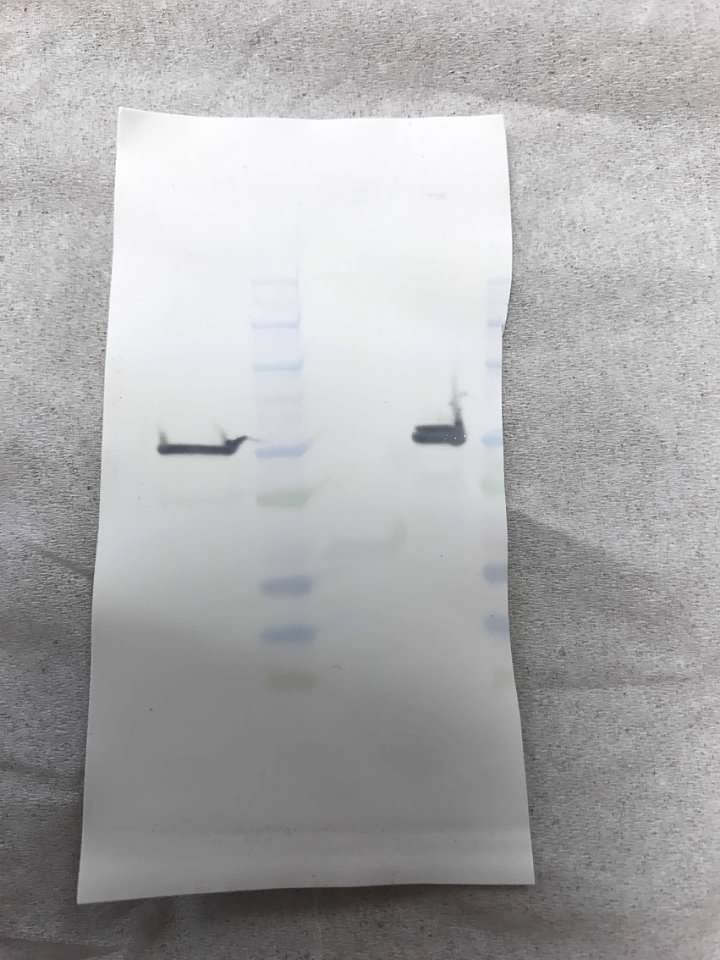

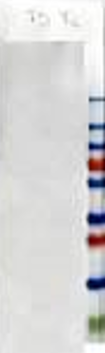

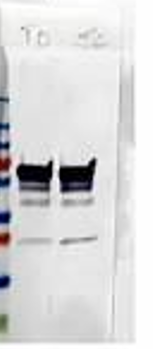


1 2 3 4 5 6 7 8 9


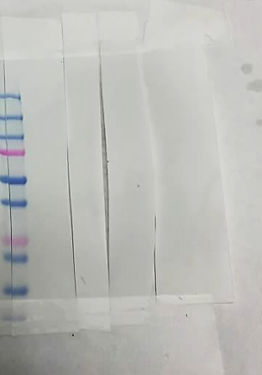

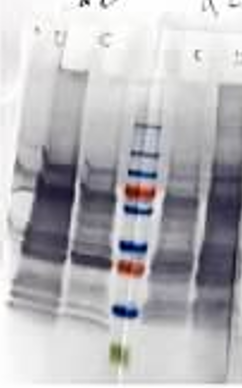


C 1 2 3 4 5 6 7 8

B

1 2 3 4 5


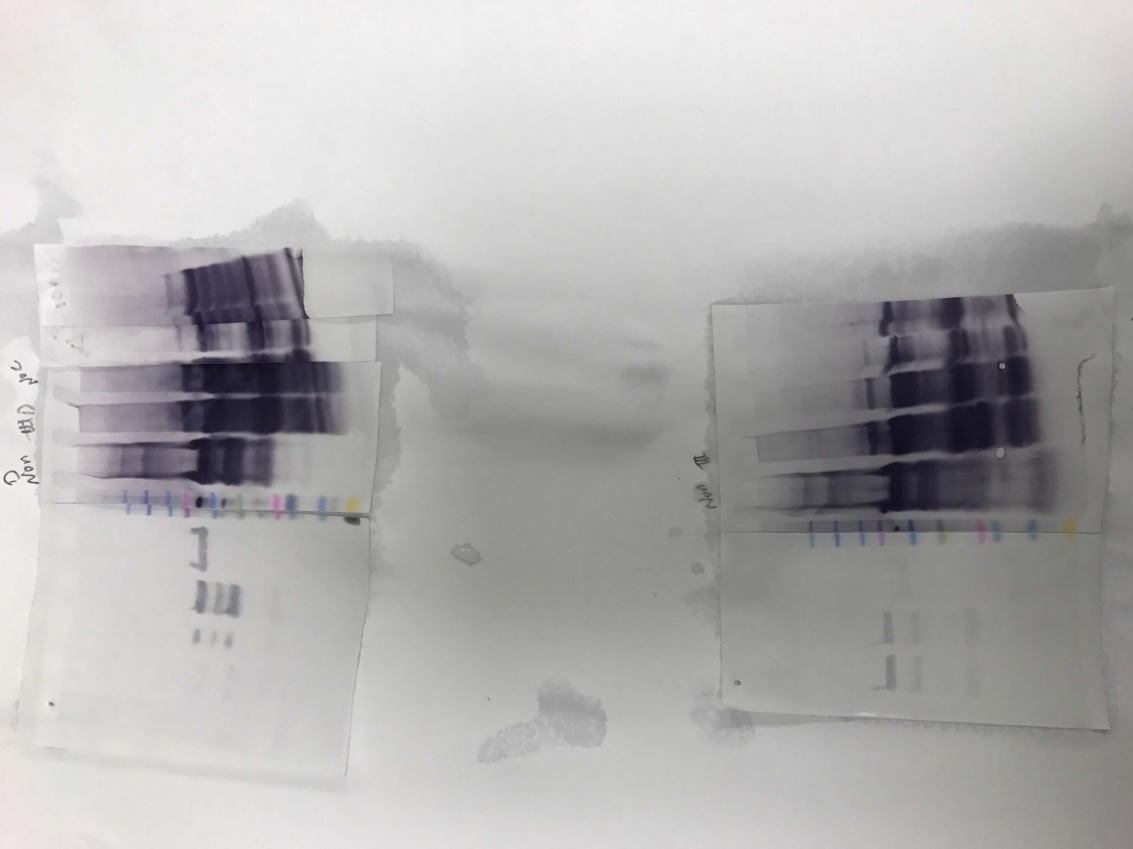


Figure S1 : Western blot identification of selected IDV proteins against A: IDV mAb:

1. Purified D/OK inactivated virus.
2. Purified D/660 inactivated virus.
3. Ladder.
4. Cleaved purified D/OK inactivated virus.
5. Cleaved purified D/660 inactivated virus.

Lane 6- 9 from lane 14-17 Fig. S3

1. IDV-HEF recombinant protein.
2. Ladder.
3. IDV-NP.
4. IDV-HEF recombinant protein.

B- Against FBS as negative control:

1. Ladder
2. Purified D/OK inactivated virus.
3. Purified D/660 inactivated virus.
4. IDV-NP.
5. IDV-HEF recombinant protein.

C: Convalescence IDV sera:

1. Purified D/OK inactivated virus.
2. Purified D/660 inactivated virus.
3. Ladder.
4. Cleaved Purified D/OK inactivated virus.
5. Cleaved Purified D/660 inactivated virus.
6. IDV-NP (Lane 8 in S2).
7. IDV-HEF recombinant protein (lane 9 in S2).
8. Ladder.


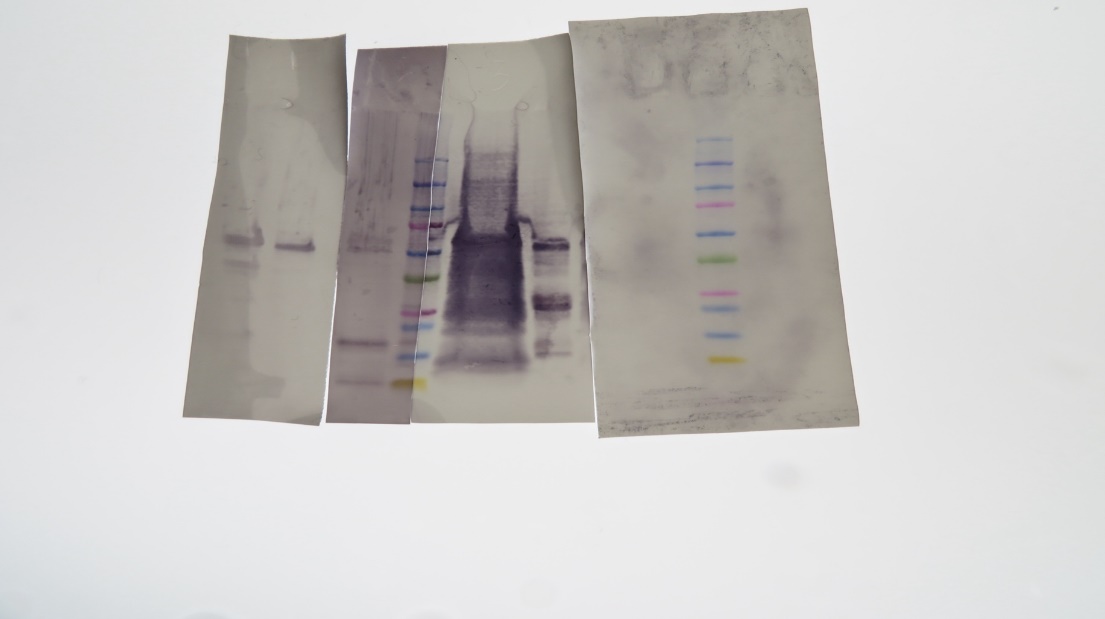

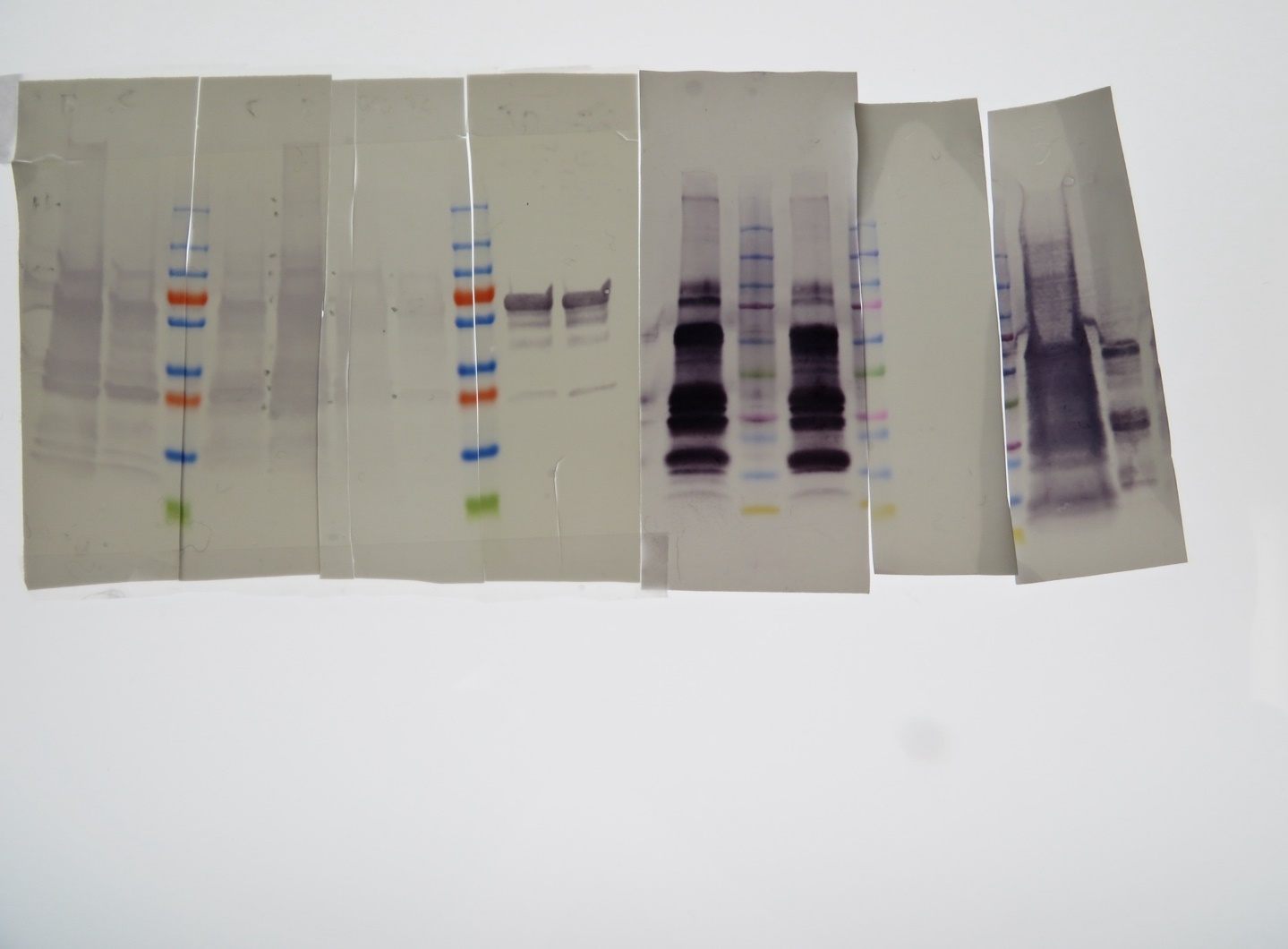


B

1 2 3 4. 5 6 7 8 9 10 11 12 13 14 15


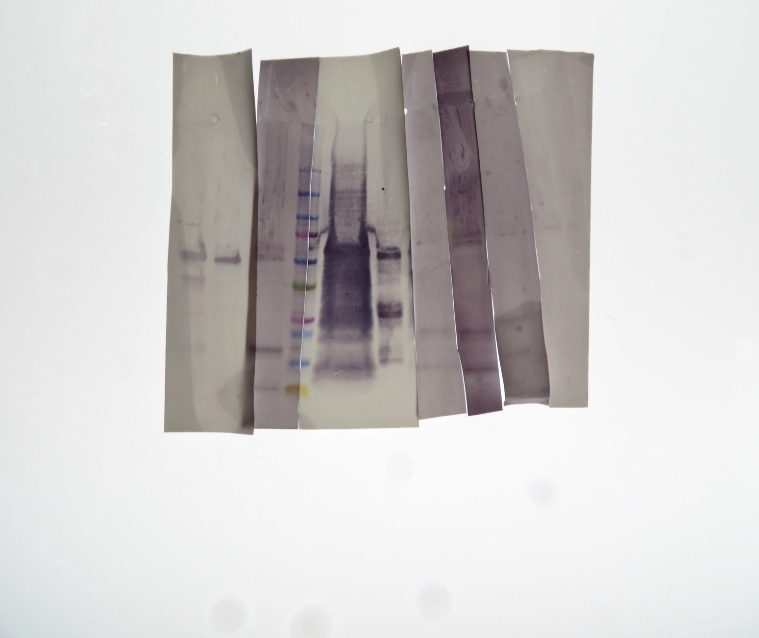

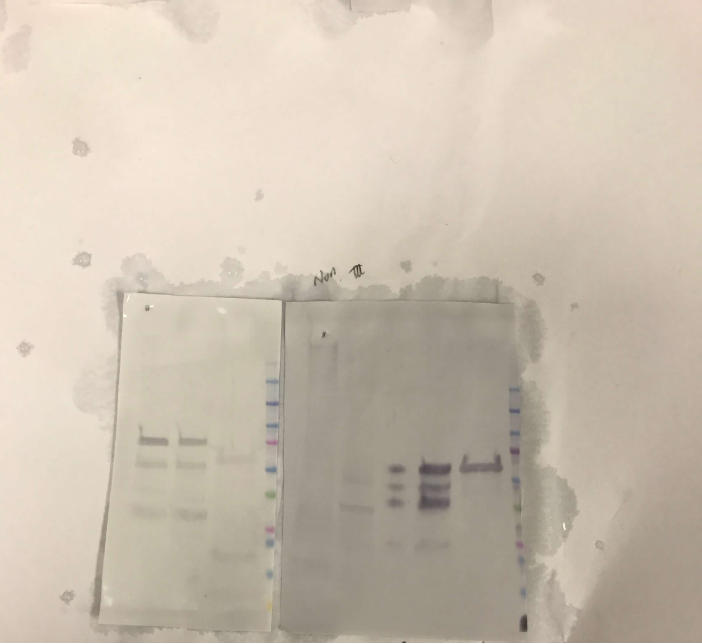


1 2 3 4. 5 6 7 8 9 10 11 12 13

A

\

Figure S2 : A- Western blot identification of selected IDV proteins using IDV mAb and IDV-convalescent sera. Lane 1-4 against IDV-mAb:

1. Cleaved purified D/OK virus.
2. Cleaved purified D/660 virus.
3. IDV-HEF recombinant protein.

Lane 5-10 against IDV-convalescent sera.

1. Ladder
2. X
3. X
4. IDV-NP
5. IDV-NP
6. IDV-HEF
7. Ladder

Lane 11-13 against FBS

B- Reactivity against IDV-mAb:

1. Cleaved purified D/OK inactivated virus.
2. Ladder
3. Cleaved purified D/660 inactivated virus.
4. Ladder.
5. Purified D/OK inactivated virus.
6. Purified D/660 inactivated virus.
7. Ladder
8. HEF recombinant protein (low conc)
9. Ladder
10. HEF recombinant protein (high conc)
11. HEF recombinant protein (medium conc)
12. NP
13. NP
14. Ladder
15. NP


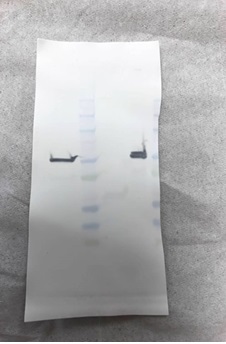

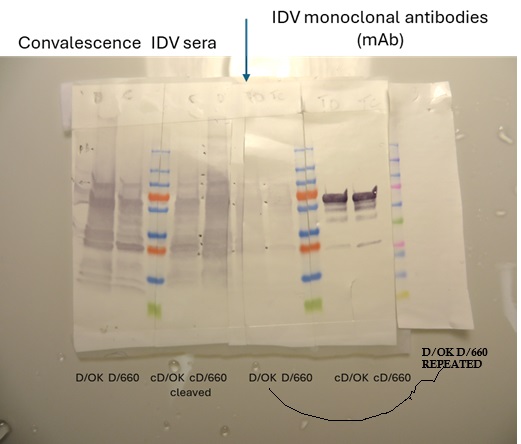


IDV mAb

14 15 16 17 L

1 2 3 4. 5 6 7 8 9 10 11 12 13

Figure S3: Western blot identification of selected IDV proteins using IDV-convalescent sera and IDV mAb.

Lane 1-5 against IDV-convalescent sera:

1. Purified inactivated D/OK virus.
2. Purified inactivated D/660 virus.
3. Ladder
4. Trypsin treated inactivated D/OK virus (cleaved).
5. Trypsin treated inactivated D/660 virus (cleaved).

Lane 6-17 against IDV-mAb.

1. Purified inactivated D/OK virus.
2. Purified inactivated D/660 virus.
3. Ladder
4. Trypsin treated inactivated D/OK virus (cleaved).
5. Trypsin treated inactivated D/660 virus (cleaved).
6. Ladder
7. Purified inactivated D/OK virus.
8. Purified inactivated D/660 virus.
9. IDV-HEF
10. Ladder
11. IDV-NP
12. IDV-HEF


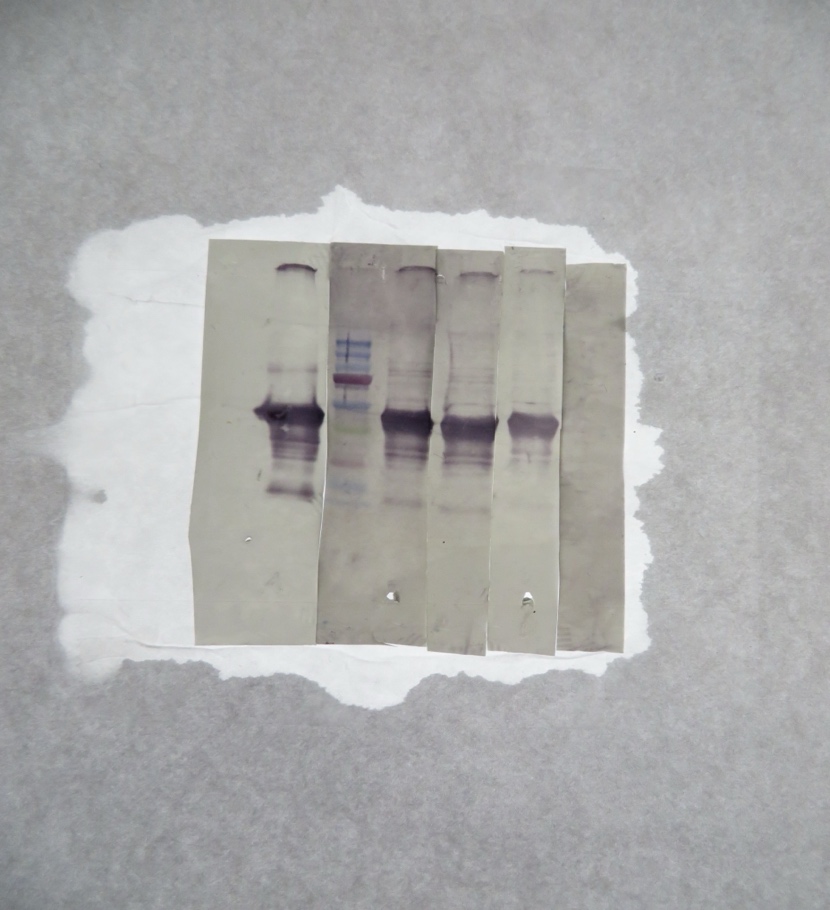


1 2 3 4 5 6 7

FIGURE S4: Another replicate for the cross-reactivity of IDV-mAb against:

1. Purified inactivated D/OK virus.
2. Trypsin treated inactivated D/OK virus (cleaved).
3. Ladder
4. Trypsin treated inactivated D/660 virus (cleaved).
5. Trypsin treated inactivated D/660 virus (cleaved).
6. IDV-HEF recombinant protein.
7. Purified inactivated D/660 virus.


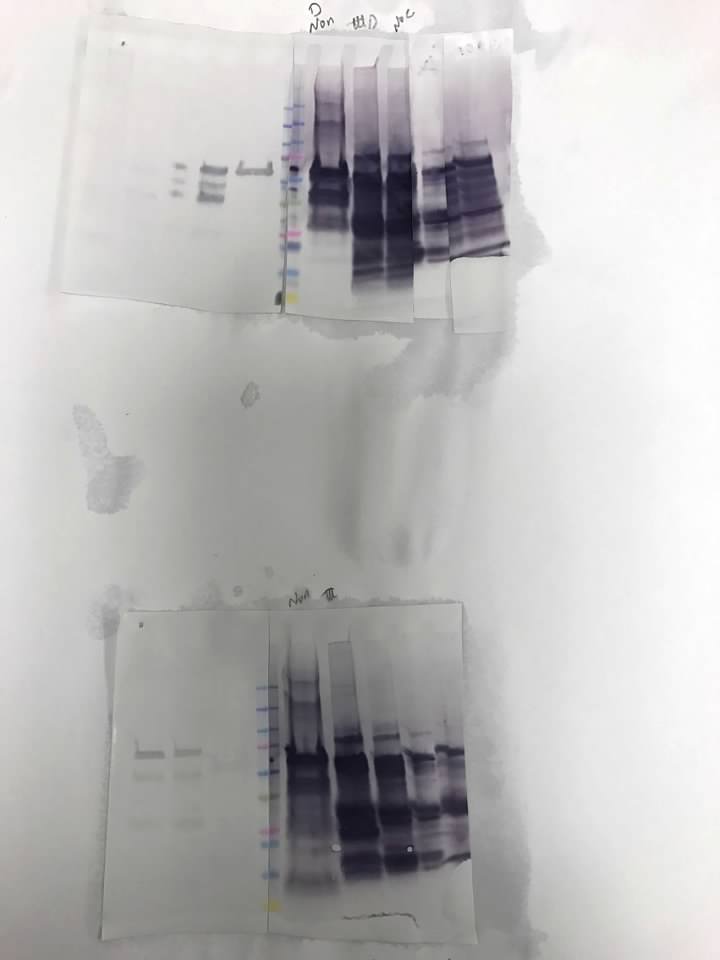


Figure S5. Cross-reactivity between two IDV different clades of D/660 and C/OK using specific polyclonal rabbit anti-sera against each clade:

1. Anti-rabbit 660
2. Ladder
3. HEF
4. Purified D/OK
5. Purified D/660
6. Cleaved D/OK
7. Cleaved D/660
8. Anti-rabbit D/OK
9. Ladder
10. HEF
11. Purified D/OK
12. Purified D/660
13. Cleaved D/OK
14. Cleaved D/660

1 2 3 4 5 6

A

B

1 2 3 4 5 6


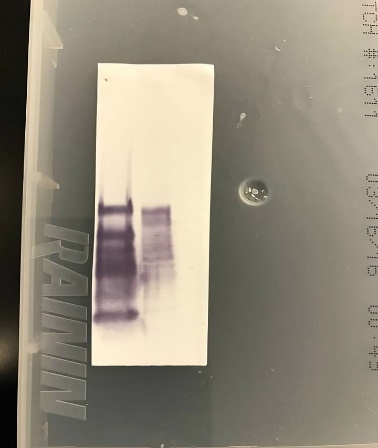


1. Anti-rabbit IDV
2. CLEAVED D/660
3. HEF
4. NP
5. HEF

C


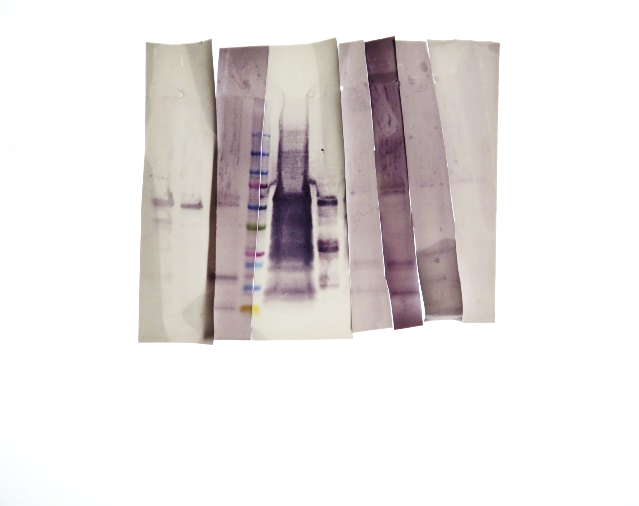

Supplement: Supplementary file 4 [file Data_Sheet_4.docx]
